# A *Drosophila* Model for EGFR-Ras and PI3K-Dependent Human Glioma

**DOI:** 10.1371/journal.pgen.1000374

**Published:** 2009-02-13

**Authors:** Renee D. Read, Webster K. Cavenee, Frank B. Furnari, John B. Thomas

**Affiliations:** 1 Molecular Neurobiology Laboratory, Salk Institute for Biological Studies, La Jolla, California, United States of America; 2 Ludwig Institute for Cancer Research, University of California San Diego, La Jolla, California, United States of America; 3 Department of Medicine, University of California San Diego, La Jolla, California, United States of America; 4 Cancer Center, University of California San Diego, La Jolla, California, United States of America; 5 Center for Molecular Genetics, University of California San Diego, La Jolla, California, United States of America; University of California San Francisco, United States of America

## Abstract

Gliomas, the most common malignant tumors of the nervous system, frequently harbor mutations that activate the epidermal growth factor receptor (EGFR) and phosphatidylinositol-3 kinase (PI3K) signaling pathways. To investigate the genetic basis of this disease, we developed a glioma model in *Drosophila*. We found that constitutive coactivation of EGFR-Ras and PI3K pathways in *Drosophila* glia and glial precursors gives rise to neoplastic, invasive glial cells that create transplantable tumor-like growths, mimicking human glioma. Our model represents a robust organotypic and cell-type-specific *Drosophila* cancer model in which malignant cells are created by mutations in signature genes and pathways thought to be driving forces in a homologous human cancer. Genetic analyses demonstrated that EGFR and PI3K initiate malignant neoplastic transformation via a combinatorial genetic network composed primarily of other pathways commonly mutated or activated in human glioma, including the Tor, Myc, G1 Cyclins-Cdks, and Rb-E2F pathways. This network acts synergistically to coordinately stimulate cell cycle entry and progression, protein translation, and inappropriate cellular growth and migration. In particular, we found that the fly orthologs of CyclinE, Cdc25, and Myc are key rate-limiting genes required for glial neoplasia. Moreover, orthologs of Sin1, Rictor, and Cdk4 are genes required only for abnormal neoplastic glial proliferation but not for glial development. These and other genes within this network may represent important therapeutic targets in human glioma.

## Author Summary

Malignant gliomas, tumors composed of glial cells and their precursors, are the most common and deadly human brain tumors. These tumors infiltrate the brain and proliferate rapidly, properties that render them largely incurable even with current therapies. Mutations in genes within the EGFR-Ras and PI3K signaling pathways are common in malignant gliomas, although how these genes specifically control glial pathogenesis is unclear. To investigate the genetic basis of this disease, we developed a glioma model in the fruit fly, *Drosophila melanogaster*. We found that constitutive coactivation of the EGFR-Ras and PI3K pathways in *Drosophila* glia gives rise to highly proliferative and invasive neoplastic cells that create transplantable tumor-like growths, mimicking human glioma. This represents a robust cell-type-specific *Drosophila* cancer model in which malignant cells are created by mutations in genetic pathways thought to be driving forces in a homologous human cancer. Genetic analyses demonstrated that EGFR-Ras and PI3K induce fly glial neoplasia through activation of a combinatorial genetic network composed, in part, of other genetic pathways also commonly mutated in human glioma. This network acts synergistically to coordinately stimulate cellular proliferation, protein translation, and inappropriate migration. Rate-limiting genes within this network may represent important therapeutic targets in human glioma.

## Introduction

Malignant gliomas, neoplasms of glial cells and their precursors, are the most common tumors of the central nervous system (CNS). These tumors typically proliferate rapidly, diffusely infiltrate the brain, and resist standard chemotherapies, properties that render them largely incurable. One key to developing more effective therapies against these tumors is to understand the genetic and molecular logic underlying gliomagenesis. The most frequent genetic lesions in gliomas include mutation or amplification of the Epidermal Growth Factor Receptor (EGFR) tyrosine kinase. Glioma-associated EGFR mutant forms show constitutive kinase activity that chronically stimulates Ras signaling to drive cellular proliferation and migration [1],[2]. Other common genetic lesions include loss of the lipid phosphatase PTEN, which antagonizes the phosphatidylinositol-3 kinase (PI3K) signaling pathway, and activating mutations in PIK3CA, which encodes the p110α catalytic subunit of PI3K [1],[2]. Gliomas often show constitutively active Akt, a major PI3K effector [1],[2]. However, EGFR-Ras or PI3K mutations alone are not sufficient to transform glial cells, rather multiple mutations that coactivate EGFR-Ras and PI3K-Akt pathways are sufficient to induce glioma [2]–[4].

Understanding the interplay of these mutations and the neurodevelopmental origins of these tumors could lead to new insights into the mechanisms of gliomagenesis. The mammalian brain contains multiple glial cell types that maintain proliferative capacities, including differentiated astrocytes, glial progenitors, and multipotent neural stem cells. EGFR-Ras and PTEN-PI3K signaling regulates many developmental processes in these cell types, particularly proliferation and self-renewal, which are also properties of glioma cells [1]. Although recent hypotheses favor that gliomas arise from multipotent stem cells, data from mouse models demonstrate that differentiated glia, glial progenitors, and stem cells can all produce gliomas in response to genetic lesions found in human gliomas [5],[6]. Thus, misregulation of these genetic pathways may confer unrestricted proliferative capacities to a range of glial cell types, but how this occurs remains unclear. While many of the same effectors are utilized by EGFR-Ras and PI3K in both glial development and cancer, constitutive activation of these pathways may deploy distinct outputs, not utilized in development, that allow particular cells to escape normal physiological cues that restrain proliferation and self-renewal. The identity of such outputs remains unclear.

With these issues in mind, we developed a *Drosophila* glioma model to facilitate genetic analysis of glial pathogenesis. *Drosophila* offers many tools for precise manipulation of cell-type-specific gene expression and dissection of multigene interactions. Most human genes, including 70% of known disease genes, have functional *Drosophila* orthologs [7]. Among the most conserved genes are components of major signal transduction pathways, including many gliomagenic genes. Recently, *Drosophila* has emerged as a model system for human neurological diseases because the CNS shows remarkable evolutionary conservation in cellular composition and neurodevelopmental mechanisms [8]. Similarly, *Drosophila* have multiple glial cell types that require the EGFR pathway for their normal development, and these cells appear homologous to mammalian glia in terms of function, development, and gene expression [9]. These similarities between flies and humans make *Drosophila* an attractive system for modeling gliomas.

## Results

### Coactivation of EGFR-Ras and PI3K in *Drosophila* Glia Causes Neoplasia

Since concurrent activation of EGFR-Ras and PI3K signaling in glial precursors induces glioma in the mouse [4], we sought to create mutant phenotypes by hyperactivation of these pathways in fly glia and glial precursors. *Drosophila* has a single functional ortholog each for EGFR(dEGFR), Raf (dRaf), PIK3CA(dp110), PTEN(dPTEN), and Akt(dAkt), and two functional orthologs for Ras(dRas85D, dRas64B) (www.flybase.org). A diagram of the specific mutant forms of dEGFR used in our assays can be found in Figure S1. We performed glial overexpression assays with the Gal4-UAS system [10], using the *repo-Gal4* driver, which gives sustained UAS-transgene expression in almost all glia, from embryogenesis through adulthood. For glial-specific RNAi, we employed UAS-dsRNA constructs [11], which we verified with phenotypic tests and/or antibody staining (see Materials and Methods). Glial morphology was visualized with membrane-localized GFP (CD8GFP) [12]. Cell number was determined with staining for Repo, a homeobox transcription factor expressed by *repo-Gal4* positive glia [13].

**Figure 1:**
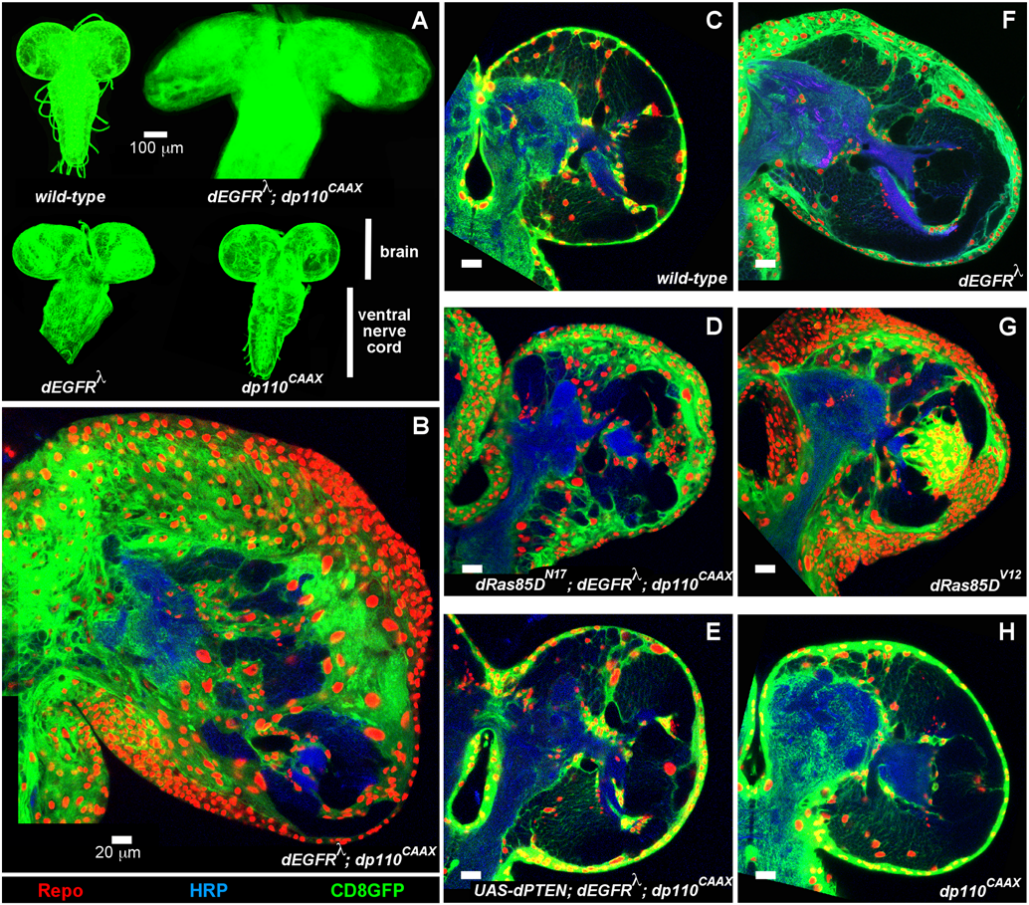
Coactivation of EGFR-Ras and PI3K in *Drosophila* glia causes neoplasia. (A) Optical projections of whole brain-ventral nerve cord complexes from late 3^rd^ instar larvae approximately 130 hr AED, displayed at the same scale. Dorsal view; anterior up. Glia are labeled with CD8GFP (green) driven by *repo-Gal4*. Each brain is composed of 2 symmetrical hemispheres attached to the ventral nerve cord (VNC). In *repo>dEGFR^λ^;dp110^CAAX^* larvae, both brain hemispheres and the VNC are enlarged and elongated relative to other genotypes. (B–H) 2 µm optical sections of larval brain hemispheres from late 3^rd^ instar larvae approximately 130 hr AED, displayed at the same scale. 20 µm scale bars. Frontal sections, midway through brains. Anterior up; midline to left. Glial cell nuclei are labeled with Repo (red). Glial cell bodies and membranes are labeled with CD8GFP (green) driven by *repo-Gal4*. In *repo>dEGFR^λ^;dp110^CAAX^* brains (B) there is a dramatic increase in number of glial nuclei relative to wild-type (C), while in *repo>dRas^N17^;dEGFR^λ^;dp110^CAAX^* (D) and *repo>PTEN; dEGFR^λ^;dp110^CAAX^* (E) brains there are fewer excess glial nuclei. In wild-type animals (C), glia extend processes throughout the entire brain, enveloping nearly every cell with glial membranes. In contrast, dEGFR^λ^;dp110^CAAX^ glia (B) show loss of fine projections and instead have compact cell bodies. Brains are counter-stained with anti-HRP (blue), which reveals neuronal fiber tracts (neuropil) at high intensity and some cell bodies of neurons and neuronal precursors at low intensity. HRP staining varies slightly according to exact plane of section, brain orientation, and mutant phenotype. Dark areas within brains contain unstained cells, which are typically optic lobe neural precursors. Genotypes: (A) Clockwise from top left: *repo-Gal4 UAS-CD8GFP/+*, *UAS-dEGFR^λ^ UAS-dp110^CAAX^/+; repo-Gal4 UAS-CD8GFP/+*, *UAS-dp110^CAAX^/+; repo-Gal4 UAS-CD8GFP/+*, *UAS-dEGFR^λ^/+; repo-Gal4 UAS-CD8GFP/+*, (B) *UAS-dEGFR^λ^ UAS-dp110^CAAX^/+; repo-Gal4 UAS-CD8GFP/+*, (C) *repo-Gal4 UAS-CD8GFP/+*, (D) *UAS-dEGFR^λ^ UAS-dp110^CAAX^/UAS-dRas85D^N17^; repo-Gal4 UAS-CD8GFP/+*, (E) *UAS-dEGFR^λ^ UAS-dp110^CAAX^/+; UAS-PTEN/+; repo-Gal4 UAS-CD8GFP/+*, (F) *UAS-dEGFR^λ^/+; repo-Gal4 UAS-CD8GFP/+*, (G) *repo-Gal4 UAS-CD8GFP/UAS-dRas85D^V12^*, (H) *UAS-dp110^CAAX^/+; repo-Gal4 UAS-CD8GFP/+*.

Glial-specific coactivation of EGFR-Ras and PI3K stimulated glial neoplasia, giving rise to CNS enlargement and malformation, neurologic defects, and late larval lethality. *repo-Gal4*-driven co-overexpression of activated dEGFR (dEGFR^λ^) and dp110 (dp110^CAAX^) induced progressive accumulation of ∼50-fold excess glia (Figure 1A and 1B) [14],[15]. dEGFR^λ^ is a constitutively active dEGFR variant in which a lambda dimerization domain replaces the extracellular domain [14] (Figure S1). Co-overexpression of combinations of dEGFR^λ^ and core components of the PI3K pathway, such as dAkt, induced phenotypes similar to *repo>dEGFR^λ^;dp110^CAAX^*, although phenotypes varied somewhat depending on strength of pathway activation and transgene expression (Figure S2 and Table S1). Dramatic glial overgrowth also occurred upon co-overexpression of constitutively active dRas (dRas85D^V12^) or its effector dRaf (dRaf^gof^) with dp110^CAAX^, dAkt, or a dPTEN^dsRNA^, which partially knocked-down dPTEN (Figure S2 and Table S1). Finally, glial overgrowth in *repo>dEGFR^λ^;dp110^CAAX^* larvae was strongly suppressed by co-overexpression of dPTEN or more moderately by dominant negative dRas85D (dRas85D^N17^) (Figure 1D and 1E), indicating that Ras activity and excess phospho-inositols are essential for neoplasia.

In contrast, glial-specific activation of the EGFR-Ras pathway alone, through overexpression of dEGFR^λ^ or Raf^gof^, induced 5–10 fold excess glia in the larval brain and later pupal lethality (Figure 1F and Table S1). dRas85D^V12^ overexpression induced approximately 5–10-fold excess glia, and these glia were smaller than wild-type or dEGFR^λ^;dp110^CAAX^ glia. (Figure 1G). dRas85D^V12^ may be more potent than dEGFR^λ^ because dRas85D^V12^ can activate endogenous PI3K signaling [16]. Overexpression of dEGFR^Elp^, a classical hypermorphic mutant form of dEGFR [17], induced excess glial proliferation and neural morphogenesis defects (Figure S2), but also caused early lethality which precluded examination of dEGFR^Elp^-dp110 interactions. As in mouse models, overexpression of wild-type dEGFR failed to induce excess glia [6],[17], and instead retarded CNS growth (Figure S2). Unlike dEGFR^λ^, dEGFR^WT^ and dEGFR^Elp^ have functional ligand-binding domains (Figure S1), and may cause additional defects by sequestering ligand otherwise required for normal development [17]. Glial-specific activation of the PI3K pathway alone, either by overexpression of dp110^CAAX^, dp110^wild-type^, dAkt, or dPTEN^dsRNA^ gave viable animals with relatively normal brains (Figure 1H, Figure S2, and Table S1). Therefore, coactivation of the EGFR and PI3K pathways synergize to produce much more severe phenotypes than would be expected if the effects of these pathways were additive.

**Figure 2:**
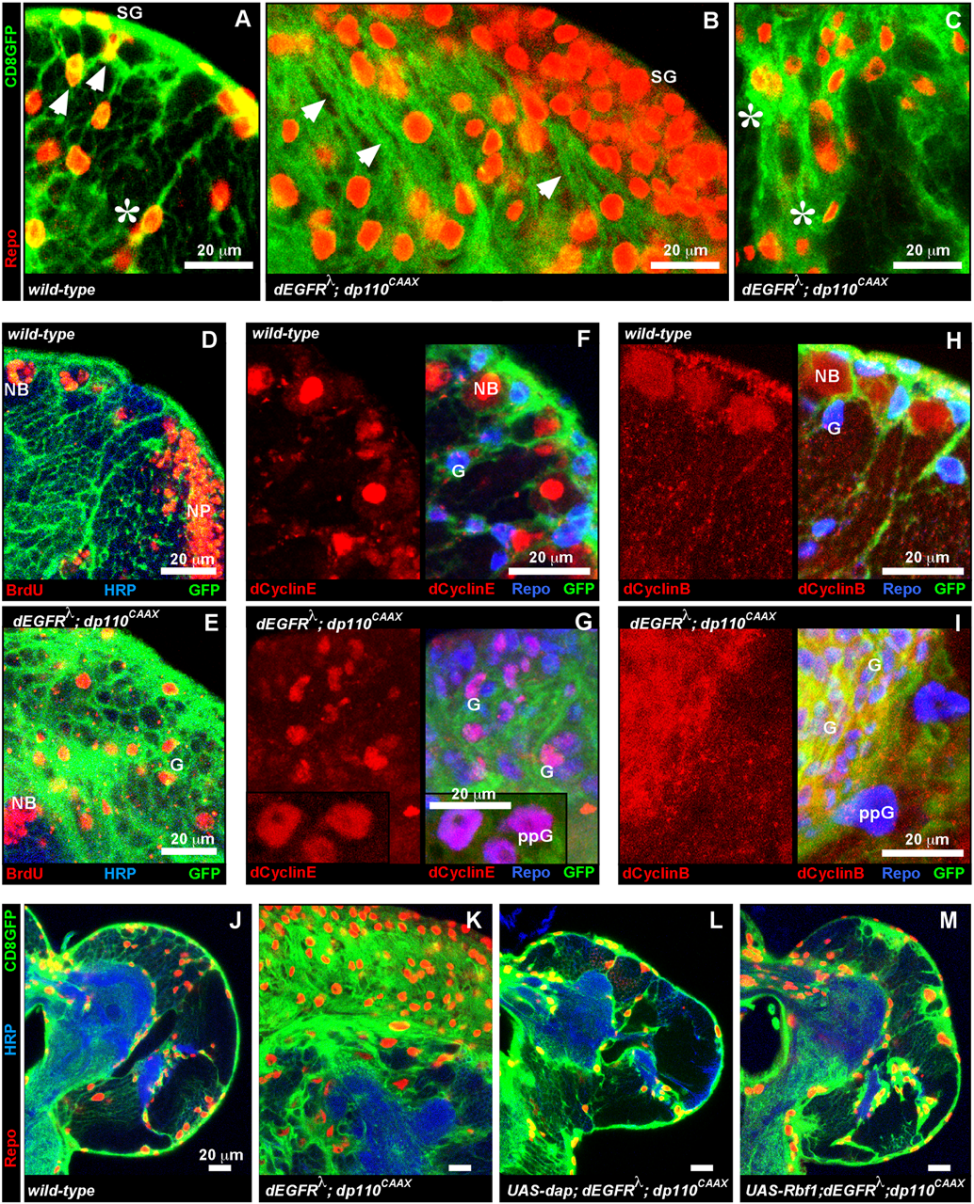
Coactivation of EGFR and PI3K cell-autonomously promotes cell cycle entry. (A–F) Close-ups of late 3^rd^ instar larval brain hemispheres. Anterior up; midline to left. 20 µm scale bars. (A–C) Glial cell nuclei are labeled with Repo (red). Glial cell bodies and membranes are labeled with CD8GFP (green) driven by *repo-Gal4*. In wild-type (A), surface glia (SG) that cover the brain form a single-cell layer of fairly flat cells whereas astrocyte-like cortex glia are often evenly spaced and form a honeycomb-like network of fine projections throughout the brain. Superficial cortex glia show stellate morphology (arrowheads), and cortex glia deeper in the brain sometimes show a radial morphology (asterisk). In *repo>dEGFR^λ^;dp110^CAAX^* brains (B,C) surface glia (SG) and superficial cortex glia (brighter GFP, arrowheads) form thick multicellular aggregations throughout the brain and often adopt a spindle-shaped morphology (arrowheads). In *repo>dEGFR^λ^;dp110^CAAX^* brains, deeper cortex glia (C) can still form fine projections, but more often lack these and create loose aggregates of abnormally shaped cells (asterisks). (D,E) BrdU labeling (red) of S-phase cells. The prominent replicative cells in normal brains (D) are neuroblasts (‘NB’) and optic lobe neural precursors (‘NP’), identified by their stereotyped position and/or staining for HRP (faint blue). The prominent replicative cells in *repo>dEGFR^λ^;dp110^CAAX^* brains (E) are glia (‘G’), which do not stain for HRP. 2 µm optical sections. (F–I) dCyclinE (F,G) and dCyclinB (H,I) expression (red) shown alone (left panels) and with Repo (blue nuclei) and CD8GFP (green, membranes/cytoplasm) glial markers (right in each panel). ‘G’ denotes representative glia, ‘NB’, representative neuroblasts. In wild-type (F, H), glia rarely expressed dCyclinE or dCyclinB, while neuroblasts (‘NB’) showed predominant expression. dCyclinE expression is primarily nuclear in dEGFR^λ^;dp110^CAAX^ glia (G), visible as purple in overlay (right panel), although not all mutant glia express dCyclinE. Inset in (G) shows dCyclinE expression in polyploid glia (‘ppG’). (I) dCyclinB expression in dEGFR^λ^;dp110^CAAX^ glia is primarily cytoplasmic, visible as yellow in overlay (right panel). ppG in *repo>dEGFR^λ^;dp110^CAAX^* brains do not express dCyclinB, as seen by the absence of yellow or purple in overlay. (F,G) 6.5 µm and (H,I) 5 µm optical projections. (J–M) 2 µm optical sections of larval brain hemispheres from late 3^rd^ instar larvae approximately 130 hr AED, displayed at the same scale. Frontal sections, midway through brains. *repo>dEGFR^λ^;dp110^CAAX^* brains (K) show a dramatic increase in number of glial nuclei (red) relative to wild-type (J). Glial cell nuclei are decreased by co-overexpression of Dap (L) or Rbf1 (M) with dEGFR^λ^;dp110^CAAX^. Glial cell bodies and membranes (green, *repo>CD8GFP* reporter) appear relatively normal in (L) and (M) compared to (J). HRP counter-stain (blue) reveals neuropil at high intensity and neuronal cell bodies at low intensity. Genotypes: (A), (D), (F), (H), and (J) *repo-Gal4 UAS-CD8GFP/+*, (B), (C), (E), (G), (I), and (K) *UAS-dEGFR^λ^ UAS-dp110^CAAX^/+; repo-Gal4 UAS-CD8GFP/+*, (L) *UAS-dEGFR^λ^ UAS-dp110^CAAX^/UAS-dap; repo-Gal4 UAS-CD8GFP/+*, (M) *UAS-dEGFR^λ^;UAS-dp110^CAAX^/+; repo-Gal4 UAS-CD8GFP/UAS-Rbf1*.

In *repo>dEGFR^λ^;dp110^CAAX^* brains, excess glia emerged in early larval stages and accumulated over 5–7 days. dEGFR^λ^;dp110^CAAX^ glia severely disrupt the normal cellular architecture of the larval brain (Figure 1A and 1B and Figure 2A–C), lose normal stellate glial morphologies (Figure 2A–C), and generate multilayered aggregations of abnormal glia throughout the brain (Figure 2A–C); in these ways dEGFR^λ^;dp110^CAAX^ glia are neoplastic [18]. Like neoplastic epithelial cells, dEGFR^λ^;dp110^CAAX^ glia ectopically expressed an active form of the matrix metalloprotease dMMP1 (Figure S3), which can confer an invasive potential [19],[20], implying that abnormal dEGFR^λ^;dp110^CAAX^ glia may be invasive within the brain. Unlike neoplastic epithelia, neoplastic neural cells, such as dEGFR^λ^;dp110^CAAX^ glia, typically retain expression of genes that regulate neural cell fate, such as Repo [21],[22].

Relative to controls, many dEGFR^λ^;dp110^CAAX^ glia showed BrdU incorporation, which marks S-phase cells (Figure 2D and 2E), indicating that neoplastic glia arise from overproliferation. *repo>dEGFR^λ^;dp110^CAAX^* animals also showed reduced BrdU in neuronal precursors (Figure 2E, data not shown), demonstrating that neoplastic glia disrupt neuronal development. The cell cycle is governed by CyclinD-Cdk4 and CyclinE-Cdk2 complexes, which phosphorylate and inactivate Rb proteins, to release E2F activators to stimulate G1-S-phase entry [23]. Kip-type (p21/p27/p57) and Ink-type cyclin-dependent kinase inhibitors antagonize proliferation by inhibiting CyclinE-Cdk2 and CyclinD-Cdk4, respectively. Cdc25 phosphatases and mitotic cyclins, including CyclinB, promote G2-M progression. Flies have single orthologs each for CyclinE, Cdk2, CyclinD, Cdk4, CyclinB, and p21/p27/p57 (Dap), E2F activators (E2F1) and two orthologs for Rb (Rbf1 and Rbf2) and Cdc25 (Stg and Twe) but no Ink ortholog [23].

dEGFR^λ^;dp110^CAAX^ glia showed ectopic expression of dCyclinE and dCyclinB (Figure 2F–I), demonstrating that EGFR and PI3K activity upregulated proteins that promote cell cycle entry and progression. High-grade human gliomas contain highly proliferative anaplastic glia and enlarged pleiomorphic polyploid glia [24]. Similarly, *repo>dEGFR^λ^;dp110^CAAX^* larvae showed accumulation of small, highly proliferative glia that strongly expressed cyclins and labeled with BrdU. *repo>dEGFR^λ^;dp110^CAAX^* larval brains also displayed abnormal polyploid glia, as assessed by DAPI staining (data not shown), and these cells typically expressed only dCyclinE but not dCyclinB, and thereby likely underwent ectopic DNA replication without mitosis (Figure 2G and 2I, data not shown). However, overexpression of dCyclinE-dCdk2, dCyclinD-dCdk4, or dE2F1-dDp complexes and/or Rbf1 knock-down did not cause neoplasia, and instead either doubled glial cell numbers or resulted in embryonic lethality (Figure S4, data not shown).

We next examined negative regulators of the cell cycle. dEGFR^λ^;dp110^CAAX^ glia expressed Rbf1, but showed little Dap, a result we also observed in wild-type glia (Figure S5). Dap inhibits dCyclinE-cdk2 complexes [25], and is transiently expressed in neural progenitors to promote cell cycle exit as they begin differentiation [26]. Dap overexpression completely suppressed *repo>dEGFR^λ^;dp110^CAAX^* glial overgrowth (Figure 2L), demonstrating that glial neoplasia is cell-autonomous and requires dCyclinE-dCdk2. Similarly, overexpressed *Rbf1* and *dCyclinE* mutations also reduced *repo>dEGFR^λ^;dp110^CAAX^* glial overproliferation (Figure 2M, data not shown). The gross neural morphogenesis defects observed in *repo>dEGFR^λ^;dp110^CAAX^* brains may be secondary to glial overproliferation since these defects were largely prevented by Dap or Rbf1 co-overexpression (Figure 2J–M). In *repo>dEGFR^λ^;dp110^CAAX^* animals, other mutant glia outside of the brain, such as peripheral glia, also became highly proliferative and invasive, and these defects, too, were corrected by Rbf1 or Dap overexpression (data not shown). In controls, Rbf1 or Dap overexpression in wild-type glia inhibited proliferation (Figure S4), reducing numbers of glia by approximately half. Together, these results suggest that *repo-Gal4* glia undergo at least one round of cell division, consistent with published studies [27],[28], and this proliferation becomes prolonged by constitutive coactivation of EGFR and PI3K signaling.

### Coactivation of EGFR and PI3K Does Not Elicit Neoplasia in All Neural Cell Types

The phenotype triggered by coactivation of EGFR and PI3K in glia is distinct from other *Drosophila* brain-overgrowth mutant phenotypes, which involve accretion of excess neurons or neuroblasts [21],[29]. *repo>dEGFR^λ^;dp110^CAAX^* cells were not transformed into neurons or neuroblasts as they lacked expression of the Elav and Miranda markers (Figure S6). Lineage-tracing with a *Su(H)-lacZ* neuroblast reporter showed that excess dEGFR^λ^;dp110^CAAX^ glia did not express LacZ, and thus are not directly derived from larval neuroblasts (data not shown). Moreover, constitutive EGFR-Ras and PI3K signaling does not elicit overgrowth in all neural cell types, as assessed with defined cell-type specific Gal4-drivers (Table S2). For example, dRas85D^V12^ overexpression in fly neurons causes defects in fate specification, patterning, and apoptosis [30],[31]. Co-overexpression of dEGFR^λ^ or dRas85D^V12^ with dp110^CAAX^ in neurons (*elav-Gal4*, *scratch-Gal4*, *OK107-Gal4*, and *Appl-Gal4*) and neuroblasts/neuronal precursors (*pros-Gal4*, *wor-Gal4*, and *1407-Gal4*) did not induce overgrowth (Figure S7, data not shown), even with increased transgene expression from Gal4 amplification [32]. In fact, broad co-overexpression of dEGFR^λ^ and dp110^CAAX^ in neuroblasts and neuronal precursors (*pros-Gal4*) reduced brain size, perhaps because signaling through these pathways stimulates precocious cell cycle exit of neuronal precursors, as in the developing eye [33]. Furthermore, transient expression of dRas85D^V12^ or dEGFR^λ^ and dp110^CAAX^ in embryonic glia (*gcm-Gal4*) also failed to promote glial overgrowth (Figure S7) [27], demonstrating that sustained activation of these pathways is required for glial overproliferation. Certain glial subtypes, such as oligodendrocyte-like neuropil glia (*Eaat1-Gal4*), some astrocyte-like cortex glia (*Nrv2-Gal4*), and peripheral perineurial glia (*gli-Gal4*) also failed to become neoplastic in response to EGFR-Ras and PI3K (Figure S7, data not shown) [34]–[36]. Therefore, neoplastic proliferation is not a uniform cellular response to EGFR and PI3K.

### Coactivation of EGFR and PI3K Inhibits Cell Cycle Exit in Glia

**Figure 3:**
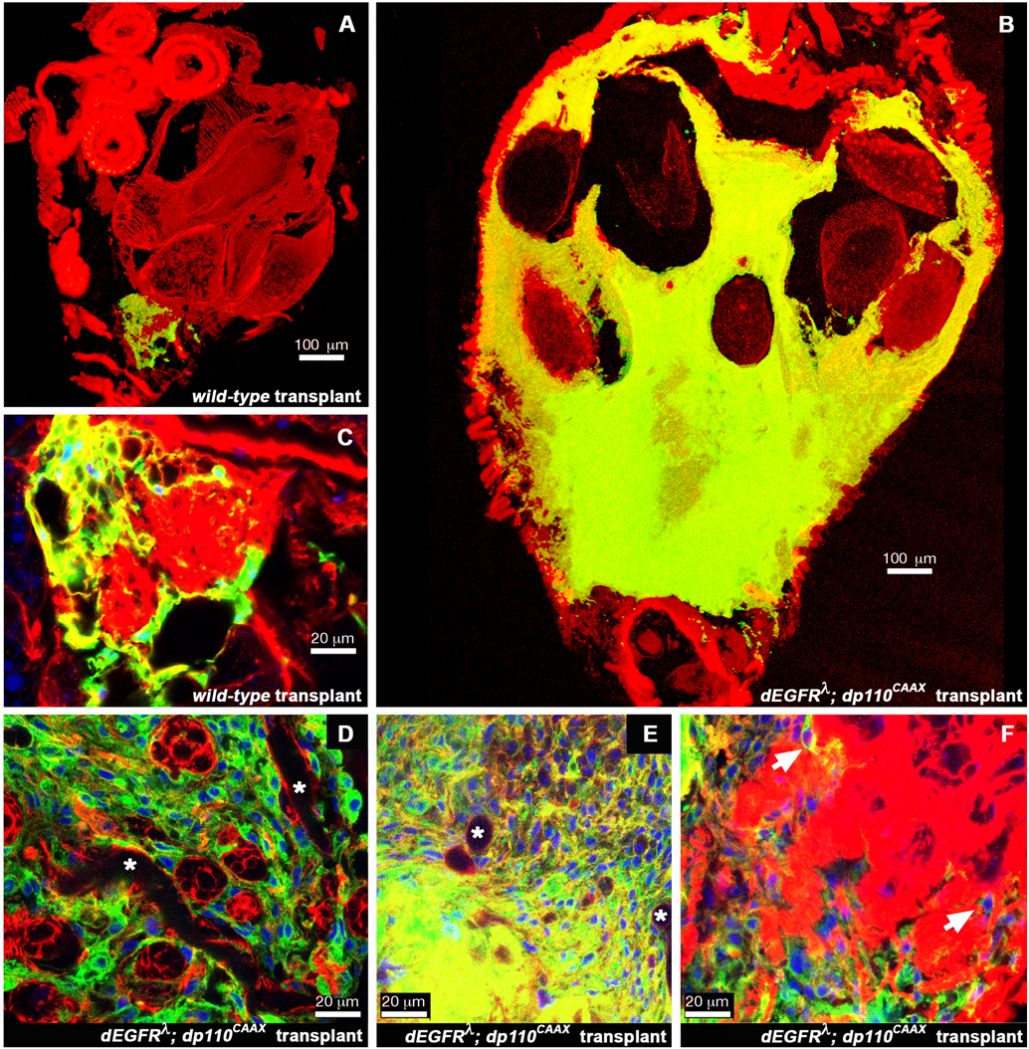
Coactivation of EGFR and PI3K inhibits cell cycle exit in glia. Larval brain fragments of the indicated genotypes were injected into the abdomens of wild-type adult hosts and grown for 3 weeks. CD8GFP (green) labels all transplanted glia within each graft. Actin (red, phalloidin) reveals abdominal anatomy. (A, B) Representative frozen sections of whole abdomens with wild-type (A) or dEGFR^λ^;dp110^CAAX^ (B) transplants, shown at the same scale. 50 µm optical projections; 100 µm scale bars. (C–F) Transplants shown at higher magnification. dEGFR^λ^;dp110^CAAX^ transplants (D–F) are rich with glial cell nuclei (Repo, in blue) relative to wild-type transplants (C). Asterisks indicate trachea embedded in dEGFR^λ^;dp110^CAAX^ transplants (D,E), visible as hollow actin-positive (red) tubules running through tissues. Arrowheads in (F) indicate dEGFR^λ^;dp110^CAAX^ glial cells invading an ovary, distinguished by its characteristic actin staining (red). 1–1.5 µm optical sections; 20 µm scale bars. Genotypes: All hosts were *w^1118^* virgin females. Transplanted glia as follows: (A) and (C) *UAS-CD8GFP/+; repo-Gal4/+*, (B), (D), (E), and (F) *UAS-dEGFR^λ^ UAS-dp110^CAAX^/+; UAS-CD8GFP/+; repo-Gal4/+*.

Since *repo>dEGFR^λ^;dp110^CAAX^* animals die in 5–7 days, we assessed the proliferative potential of mutant glia using an abdominal transplant assay, a classic test of tumorigencity in flies [37]. Brain fragments from *repo>dEGFR^λ^;dp110^CAAX^* and wild-type larvae were transplanted into young adults. Wild-type transplants grew and survived over 1–6 weeks, but produced few glia (Figure 3A and 3C). dEGFR^λ^;dp110^CAAX^ mutant glia survived and proliferated into massive tumors that filled the hosts' abdomens, often causing premature death (Figure 3B). Tumors were composed of small glial cells with little cytoplasm (Figure 3D–F). Tumors also contained trachea embedded throughout their mass (Figure 3D and 3E and Video S1), suggesting that tumors stimulated growth of new trachea or enveloped existing trachea, perhaps in a process akin to tumor angiogenesis. The leading edges of the tumors harbored individual cells invading nearby tissues, such as the ovary (Figure 3F and Video S2), which is consistent with the ectopic expression of active dMMP1 observed in dEGFR^λ^;dp110^CAAX^ glia in the larval brain (Figure S3). However, some tissues, such as the gut, did not contain metastases, implying some degree of selective invasion. Thus, once unconstrained by the larval life cycle, dEGFR^λ^;dp110^CAAX^ glia fail to exit the cell cycle, continue to proliferate, and form highly invasive tumors, all properties of human cancer cells.

### Coactivation of EGFR-Ras and PI3K Creates Invasive Neoplastic Glia

To explore the invasive potential of mutant glia, we used FLP-FRT clonal analysis, a technique in which discrete clones of mutant cells are induced in otherwise normal tissues, a situation analogous to somatic tumorigenesis. We used a heat-shock-driven FLP-recombinase to catalyze mitotic recombination between FRT-bearing chromosomes such that a daughter cell (and all of its clonal progeny) initiated expression of GFP and UAS-containing transgenes only in *repo-Gal4*-expressing cells [12]. Clones were induced late in development, from mitotic founder cells, and were examined in adults. We could not definitively determine if clones were derived from single cell events since our study of these clones was retrospective, but given the frequency of control clone induction, many mutant clones likely originated from single cells.

**Figure 4:**
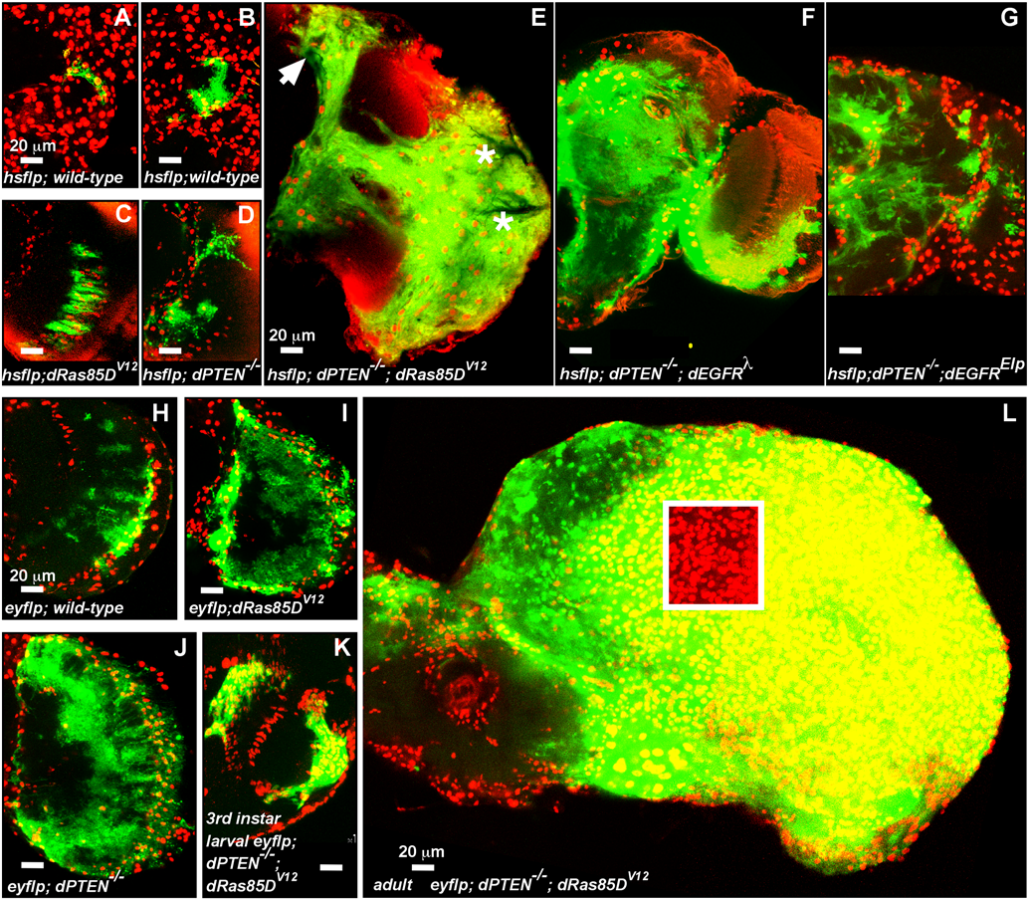
Coactivation of EGFR-Ras and PI3K creates invasive neoplastic glia. FLP/FRT clones of *repo-Gal4*-expressing glia within whole adult brains, all displayed at the same scale. CD8GFP (green) labels the cell bodies and membranes of glial clones derived by FLP/FRT mitotic recombination (see text). Repo (red) labels all glial cell nuclei, in both clones and surrounding normal tissue. Red flare is background flourescence in (C–F). Frontal sections; anterior up; midline to left. 20 µm scale bars. (A–G) hs-FLP clones from approximately 2 week-old adults, heat-shocked as late pupae or young adults. (A–B) GFP-positive wild-type control clones composed of 1–3 cells with normal stellate morphology. (C) a *dRas85D^V12^-*expressing clone in the same brain region as (D,E). *dRas85D^V12^* clones typically display the most proliferation among single mutant clones, containing approximately 2–5× more cells than controls. (D) a *dPTEN^−/−^* clone with abnormal morphology and punctate membrane-bound CD8GFP. (E) a *dRas85D^V12^; PTEN^−/−^* double mutant clone, composed of hundreds of cells that have overtaken the optic lobe and invaded the central brain (arrow), extending through the entire depth of the brain (Video S3). Asterisks demark optically opaque tracheal branches. (F,G) *dEGFR^λ^;dPTEN^−/−^* and *dEGFR^Elp^;dPTEN^−/−^* clones, which typically have less cellular density than *dRas85D^V12^; PTEN^−/−^* clones. 5–10 µm optical projections. (H–L) ey-FLP clones derived from a population of *ey-FLP* and *repo-Gal4*-expressing mitotic cells. (H–J,L) 8.5 µm confocal optical projections of whole optic lobes from brains of similarly aged adults. Both *dRas85D^V12^* (I) and *dPTEN^−/−^* (J) clones contain 2–5-fold more cells than wild-type controls (H). *dRas85D^V12^; PTEN^−/−^* double mutant clones (L) form large glial tumors. White-bordered cut-out shows the high density of glial cell nuclei (red) in *dRas85D^V12^; PTEN^−/−^* double mutant clones (L). (K) shows an earlier stage *dRas85D^V12^; PTEN^−/−^* clone in the larval brain, where *dRas85D^V12^; PTEN^−/−^* clones begin as clusters of tens of cells, and grow to become large clones of hundreds of cells by early adulthood, as in (L). Genotypes: (A) and (B) *hs-flp1/+;FRT^G13^ tubGal80/FRT^G13^ UAS-CD8GFP; repo-Gal4/+*, (C) *hs-flp1/+; FRT^G13^ tubGal80/FRT^G13^ UAS-CD8GFP; repo-Gal4/UAS-dRas85D^V12^*, (D) *hs-flp1/+; FRT^40A^ tubGal80/FRT^40A^ PTEN^2L117^; repo-Gal4 UAS-CD8GFP/+*, (E) *hs-flp1/+; FRT^40A^ tubGal80/FRT^40A^ PTEN^2L117^; repo-Gal4 UAS-CD8GFP/UAS-dRas85D^V12^*, (F) *hs-flp1/+; FRT^40A^ tubGal80/FRT^40A^ PTEN^2L117^; repo-Gal4 UAS-CD8GFP/UAS-dEGFR^λ^*, (G) *hs-flp1/+; FRT^40A^ tubGal80/FRT^40A^ PTEN^2L117^; repo-Gal4 UAS-CD8GFP/UAS-dEGFR^Elp^*, (H) *ey-flp/+; FRT^40A^ tubGal80/FRT^40A^; repo-Gal4 UAS-CD8GFP/+*, (I) *ey-flp/+; FRT^G13^ tubGal80/FRT^G13^ UAS-CD8GFP; repo-Gal4/UAS-dRas85D^V12^*, (J) *ey-flp/+; FRT^40A^ tubGal80/FRT^40A^ PTEN^2L117^; repo-Gal4 UAS-CD8GFP/+*, (K and L) *ey-flp/+; FRT^40A^ tubGal80/FRT^40A^ PTEN^2L117^; repo-Gal4 UAS-CD8GFP/UAS-dRas85D^V12^*.

In wild-type controls, we observed clones in 68% of brains examined (N = 149). Of the brains with clones, 75% had 1–3 clones, and 83% of these clones consisted of 1–3 cells of the same glial subtype (Figure 4A and 4B). Glial clones overexpressing dRas85D^V12^, dEGFR^λ^, or dEGFR^Elp^ alone typically contained 2-fold more cells than wild-type (dRas85D^V12^ shown in Figure 4C). To examine PI3K signaling, we used a *dPTEN* null allele, which became homozygous in FLP-FRT clones. *dPTEN^−/−^* glia did not overgrow, but did show aberrant cytoplasmic projections (Figure 4D), perhaps reflecting dPTEN function in the cytoskeleton [38].

To coactivate EGFR-Ras and PI3K in glial clones, dRas85D^V12^, dEGFR^Elp^, or dEGFR^λ^ was overexpressed within *dPTEN^−/−^* glia, using *repo-Gal4*. We observed overgrown and invasive *dRas85D^V12^;dPTEN^−/−^* and *dEGFR^Elp^;dPTEN^−/−^* clones (Figure 4E–G). *dEGFR;dPTEN^−/−^* clones were less affected (Figure 4F and 4G), consistent with the larval overexpression of dRas85D^V12^ giving more severe growth phenotypes. Tumor-like overgrowth was only observed in *dEGFR-dRas85D;dPTEN^−/−^* cells, illustrating that chronic EGFR-Ras activation and PTEN loss can cause cell-autonomous over-proliferation. Cells from these double mutant clones appeared to invade the brain, typically following fiber tracts, and sometimes induced the formation of trachea (Figure 4E). Tumor-like growths of *dEGFR-dRas85D;dPTEN^−/−^* cells often penetrated deep into the brain, as exemplified by Videos S3 and S4 which show an animated 88 µm thick confocal z-stack of the *dRas85D^V12^*;*dPTEN^−/−^* clone in Figure 4E compared to a 16 µm thick z-stack of a wild-type control clone in Figure 4A. These phenotypes were reminiscent of invasion and angiogenesis in human gliomas [24]. We more commonly observed smaller *dEGFR-dRas85D;dPTEN^−/−^* clones composed of relatively differentiated, enlarged glia with diffusely invasive projections (Figure 4G); these clones likely derive from glia that differentiated prior to achieving sufficient EGFR-Ras transgene expression, and are consistent with findings that not all glial subtypes become neoplastic.

Since neoplastic larval glia were concentrated in the outer anterior central brain and developing optic lobe, they may be derived from glial progenitor cells present in these regions [27],[28],[39]. To create clones from a discrete subpopulation of glial progenitors, we used an eyeless(ey)-promoter driven FLP-recombinase (ey-FLP), which is active in ey-expressing glial progenitors in the optic lobe [27]. Single mutant ey-FLP clones of *dPTEN^−/−^,* dRas85D^V12^, or dEGFR^Elp^ cells contained a modest number of excess and abnormal glia relative to wild-type controls (Figure 4H–J and Figure S8). In contrast, the double mutant *dRas85D^V12^;dPTEN^−/−^*, *dEGFR^Elp^;dPTEN^−/−^*, or *dEGFR^λ^;dPTEN^−/−^* ey-FLP clones, which emerge as approximately tens of cells in 3^rd^ instar larval brains (Figure 4K), became large invasive tumors composed of hundreds to thousands of cells in adults (Figure 4L and Figure S8).

### EGFR;PI3K Induces Glial Neoplasia via Pnt and Stg Expression

To address the function of EGFR-Ras and PI3K in glioma, we analyzed the genetic basis of glial pathogenesis in our *repo>dEGFR^λ^;dp110^CAAX^* model, as this model shows robust neoplasia, similarity to human tumor genotypes, and sensitivity to *dEGFR* and *dPTEN* gene dosage (data not shown).

**Figure 5:**
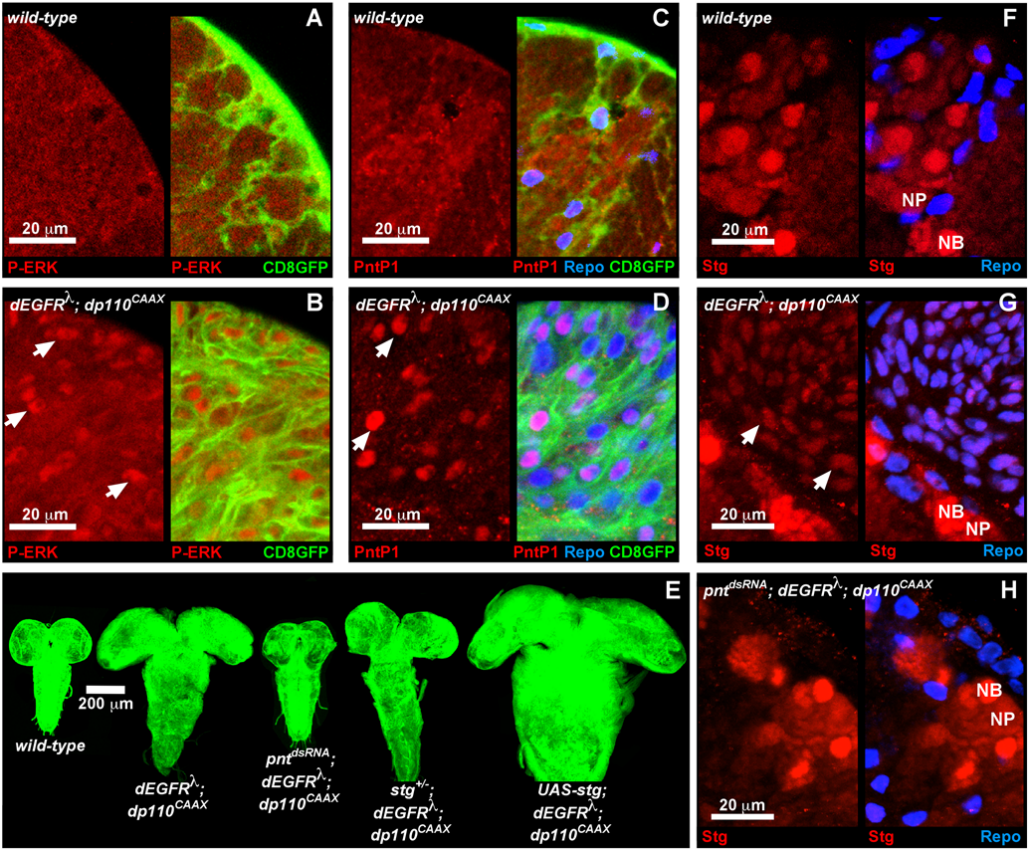
EGFR;PI3K induces glial neoplasia via Pnt and Stg expression. (A–D,F–H) late 3^rd^ instar larval brain hemispheres. Staining patterns are shown alone (left panels) and with overlaid *repo-Gal4*-driven CD8GFP (green) and/or Repo (blue) glial markers (right panels). Close-ups of medial anterior regions; anterior up; midline to left. 20 µm scale bars. (A,B): Diphospho-ERK (P-ERK, red) staining in wild-type (A) and *repo>dEGFR^λ^;dp110^CAAX^* (B) brains. High levels of nuclear ERK (arrows) are present in dEGFR^λ^;dp110^CAAX^ glia, but not in wild-type glia. Glial cell bodies are labeled green in overlay (right panels). 2 µm optical sections. (C,D): PntP1 expression (red) in wild-type (C) and *repo>dEGFR^λ^;dp110^CAAX^* (D) brains. Intense PntP1 expression in dEGFR^λ^;dp110^CAAX^ glial nuclei (arrows) is coincident with Repo and visible as purple nuclei in overlay (D, right panel). 3 µm optical projections. (E) Optical projections of whole brain-ventral nerve cord complexes from late 3^rd^ instar larvae of the indicated genotype, approximately 130 hr AED, all matched in scale. CD8GFP (green) expression in all *repo-Gal4-*expressing glia reveals structure of the entire CNS. 200 µm scale bar. (F–H): Stg expression (red) in wild-type (F), *repo>dEGFR^λ^;dp110^CAAX^* (G), and *repo>pnt^dsRNA^;dEGFR^λ^;dp110^CAAX^* (H) brains. Stg expression in glia is visible as purple nuclei in overlay (right panels). *repo>pnt^dsRNA^;dEGFR^λ^;dp110^CAAX^* glia (H) show reduced Stg compared to *repo>dEGFR^λ^;dp110^CAAX^* glia (G, arrows indicate some Stg-expressing glia). Intense Stg expression (red only) in neuroblasts (‘NB’) and ganglion mother cell neuronal progenitors (‘NP’) is visible in all panels. Stg is visualized with the *stg^CB03726^* protein trap, a viable *stg* allele that contains a GFP-expressing exon [68] and reflects the observed patterns of endogenous *stg* expression (data not shown). 5 µm optical projections. Genotypes: (A), (C), and (F) *repo-Gal4 UAS-CD8GFP/+*, (B), (D), and (G) *UAS-dEGFR^λ^ UAS-dp110^CAAX^/+; repo-Gal4 UAS-CD8GFP/+*, (E) from left to right: *repo-Gal4, UAS-CD8GFP/+*, *UAS-dEGFR^λ^ UAS-dp110^CAAX^/+; repo-Gal4 UAS-CD8GFP/+*, *UAS-dEGFR^λ^ UAS-dp110^CAAX^/+; repo-Gal4 UAS-CD8GFP/UAS-pnt^dsRNA^*, *UAS-dEGFR^λ^ UAS-dp110^CAAX^/+; repo-Gal4 UAS-CD8GFP/stg^01235^*, *UAS-dEGFR^λ^ UAS-dp110^CAAX^/+; UAS-stg/+; repo-Gal4 UAS-CD8GFP/+*, (F) *stg^CB03726^/repo-Gal4 UAS-CD8GFP*, (G) *UAS-dEGFR^λ^ UAS-dp110^CAAX^/+; stg^CB03726^/repo-Gal4 UAS-CD8GFP*, (H) *UAS-dEGFR^λ^ UAS-dp110^CAAX^/+; stg^CB03726^ UAS-pnt^dsRNA^/repo-Gal4 UAS-CD8GFP*.

EGFR-Ras signaling can promote proliferation through Erk kinase-mediated induction of nuclear targets. In *repo>dEGFR^λ^;dp110^CAAX^* brains, mutant glia showed high levels of nuclear, activated di-phospho-Erk relative to wild-type glia in control brains (Figure 5A and 5B). In flies, Erk activity can induce expression of PntP1, an ETS-family transcription factor encoded by the *pointed* (*pnt*) locus [40],[41]. PntP1, which is expressed in embryonic glia and is required for their normal development [40], was upregulated in dEGFR^λ^;dp110^CAAX^ glia (Figure 5C and 5D). High levels of PntP1 can be detected normally in neuronal progenitors (data not shown), suggesting that it promotes a proliferative progenitor state. In developing eye tissue, EGFR-Ras-Erk signaling induces Pnt proteins to stimulate G2-M cell cycle progression through direct upregulation of Stg (Cdc25 ortholog) expression [41]. Glial-specific RNAi knock down of *pnt* reduced Stg expression and completely suppressed dEGFR^λ^;dp110^CAAX^ neoplasia (Figure 5E–H), demonstrating that Pnt proteins are required for both Stg expression and neoplastic overproliferation in dEGFR^λ^;dp110^CAAX^ glia. Notably, in *repo>pnt^dsRNA^;dEGFR^λ^;dp110^CAAX^* brains, glia maintained their fate, as evidenced by *repo-Gal4* and Repo expression.

Stg itself was rate limiting for glial neoplasia. Reduction of *stg* with a mutation or a *stg^dsRNA^* partially suppressed the *repo>dEGFR^λ^;dp110^CAAX^* phenotype, whereas overexpressed Stg synergistically enhanced neoplasia (Figure 5E and Figure S9, data not shown). In contrast, Stg overexpression alone increased glial cell numbers approximately 2-fold, and could not induce neoplasia when combined with PI3K effectors (data not shown). Thus, dEGFR^λ^;dp110^CAAX^ induces neoplasia via coordinated stimulation of G1-S entry through dCyclinE, and G2-M progression through Stg, both of which are EGFR-Ras dependent outputs [41],[42].

### EGFR;PI3K Glial Neoplasia Requires Akt and Tor Activation and FoxO Inactivation

**Figure 6:**
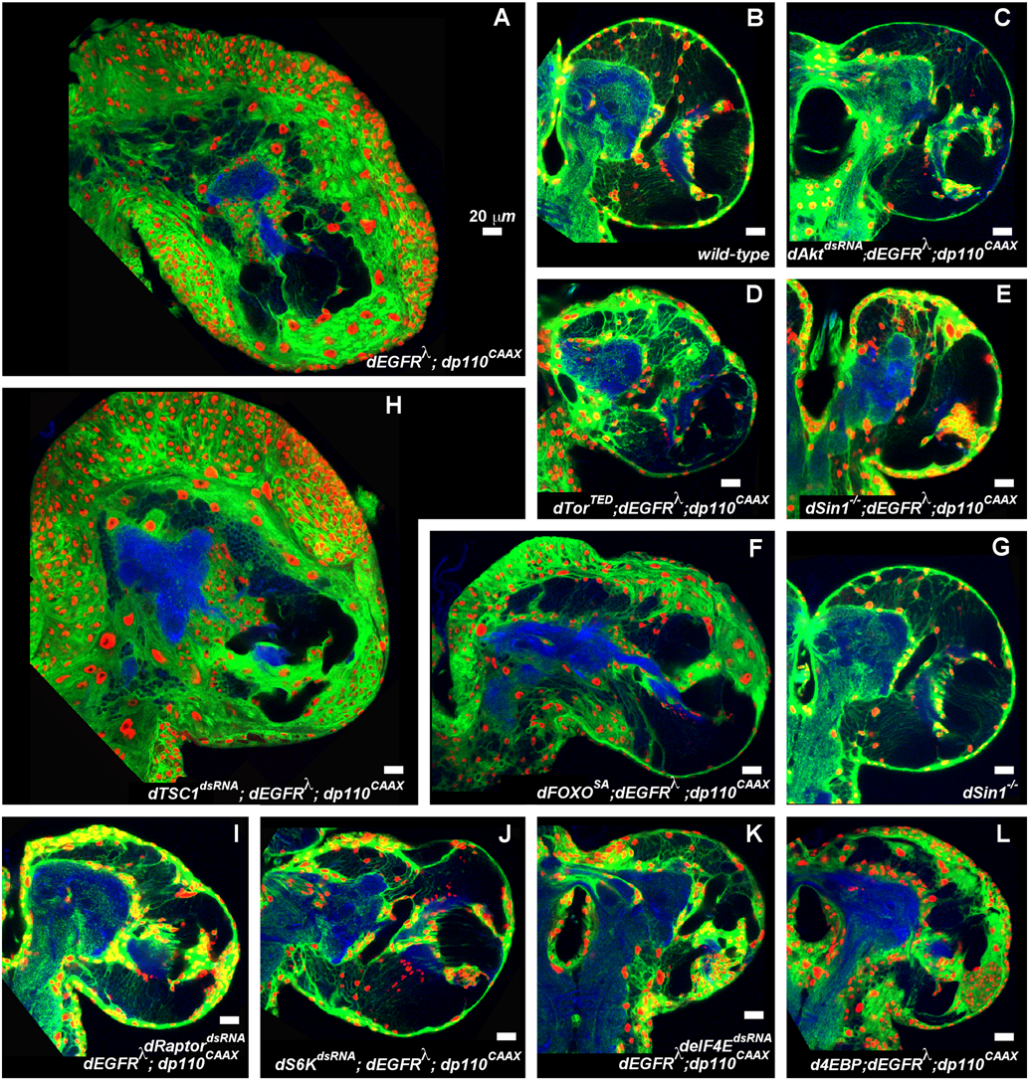
EGFR;PI3K glial neoplasia requires Akt and Tor activation and FoxO inactivation. 2 µm optical sections of larval brain hemispheres from late 3^rd^ instar larvae, all approximately 130 hr AED, displayed at the same scale. 20 µm scale bars. Frontal sections, midway through brains. Anterior up; midline to left. Glial cell nuclei are labeled with Repo (red); glial cell bodies and membranes are labeled with CD8GFP (green) driven by *repo-Gal4*. An anti-HRP counter-stain (blue) reveals neuropil (neuronal fibers) at high intensity and neuronal cell bodies at low intensity. HRP staining can vary slightly according to exact plane of section and brain orientation. *repo>dAkt^dsRNA^;dEGFR^λ^;dp110^CAAX^* (C), *repo>dTor^TED^;dEGFR^λ^;dp110^CAAX^* (D), *dSin1^−/−^;repo>dEGFR^λ^;dp110^CAAX^* (E), *repo>dRaptor^dsRNA^;dEGFR^λ^;dp110^CAAX^* (I), *repo>dS6K^dsRNA^;dEGFR^λ^;dp110^CAAX^* (J), *repo>deIF4E^dsRNA^;dEGFR^λ^;dp110^CAAX^* (K), and *repo>d4EBP;dEGFR^λ^;dp110^CAAX^* (L) brains showed near normal (B) numbers of glial nuclei (red) relative to *repo>dEGFR^λ^;dp110^CAAX^* brains (A). *repo>dFoxO^SA^;dEGFR^λ^;dp110^CAAX^* (F) shows partial reduction in number of glial nuclei (red) relative to *repo>dEGFR^λ^;dp110^CAAX^* brains (A). *repo>dTSC1^dsRNA^;dEGFR^λ^;dp110^CAAX^* brains (H) show increased numbers of glial nuclei relative to *repo>dEGFR^λ^;dp110^CAAX^* brains (A). *dSin1^−/−^* (G) brains displayed normal numbers of glia (B). Genotypes: (A) *UAS-dEGFR^λ^ UAS-dp110^CAAX^/+; UAS-lacZ^nls^/+; repo-Gal4 UAS-CD8GFP/+*, (B) *repo-Gal4 UAS-CD8GFP/+*, (C) *UAS-dEGFR^λ^ UAS-dp110^CAAX^/+; repo-Gal4 UAS-CD8GFP/UAS-dAkt^dsRNA^*, (D) *UAS-dEGFR^λ^ UAS-dp110^CAAX^/+; UAS-dTor^TED^/+; repo-Gal4 UAS-CD8GFP/+*, (E) *UAS-dEGFR^λ^ UAS-dp110^CAAX^/+; dSin1^ e03756/l(2)Bsc11^; repo-Gal4 UAS-CD8GFP/+*, (F) *UAS-dEGFR^λ^ UAS-dp110^CAAX^/+; UAS-dFoxO^SA^/+; repo-Gal4 UAS-CD8GFP/+*, (G) *dSin1^e03756/l(2)Bsc11^; repo-Gal4 UAS-CD8GFP/+*, (H) *UAS-dEGFR^λ^ UAS-dp110^CAAX^/+; repo-Gal4 UAS-CD8GFP/UAS-dTSC1^dsRNA^*, (I) *UAS-dEGFR^λ^ UAS-dp110^CAAX^/+; UAS-dRaptor^dsRNA^/+; repo-Gal4 UAS-CD8GFP/+*, (J) *UAS-dEGFR^λ^ UAS-dp110^CAAX^/UAS-dS6K^dsRNA^; repo-Gal4 UAS-CD8GFP/+*, (K) *UAS-dEGFR^λ^ UAS-dp110^CAAX^/+; repo-Gal4 UAS-CD8GFP/UAS-deIF4E^dsRNA^*, (L) *UAS-dEGFR^λ^ UAS-dp110^CAAX^/+; UAS-d4EBP/+; repo-Gal4 UAS-CD8GFP/+*.

We sought to determine which PI3K effectors contribute to the *repo>dEGFR^λ^;dp110^CAAX^* phenotype. Genetic reduction of dAkt, a major target of PI3K signaling, with a *dAkt^dsRNA^* or a mutant allele strongly suppressed *repo>dEGFR^λ^;dp110^CAAX^* glial neoplasia (Figure 6A–C, data not shown). Therefore, Akt is necessary for the outcome of EGFR-PI3K coactivation. Many Akt effectors are implicated in glioma, and we tested orthologs of these loci in our model (Table S3).

Tor, a kinase that promotes cell growth and proliferation, is a key Akt target. In glioma models, coactivation of EGFR-Ras and PI3K stimulates Tor, and in humans, Tor activity is correlated with poor patient prognosis [43],[44]. We tested the single *Drosophila* Tor ortholog, dTor, by genetically reducing dTor activity in *repo>dEGFR^λ^;dp110^CAAX^* larvae with a viable combination of hypomorphic *dTor* alleles or by co-overexpression of dominant negative dTor 45,46. Both of these manipulations reduced glial overgrowth (Figure 6D, data not shown).

In flies and mammals, Tor exists in two different signaling complexes, TORC1 and TORC2. TORC2, a complex including Tor and the Sin1 and Rictor regulatory proteins, directly phosphorylates Akt, creating a positive feedback loop that fully activates Akt [47]. In mouse, *Sin1* and *Rictor* mutants die early due to extraembryonic defects, but *dSin1* and *dRictor* mutant flies are viable as homozygous nulls, allowing us to remove TORC2 function genetically [47],[48]. *dSin1^−/−^; repo>dEGFR^λ^;dp110^CAAX^* larval brains showed a near-wild type phenotype (Figure 6E). Results were similar with a *dRictor* null allele and a *dSin1^dsRNA^* (data not shown). *dSin1^−/−^* and *dRictor^−/−^* mutants display reduced Akt-dependent phosphorylation and inactivation of dFoxO [49], the single *Drosophila* ortholog of FoxO transcription factors. This suggests that TORC2 loss might antagonize glial neoplasia through dFoxO upregulation. However, excess dFoxO^SA^, which is resistant to dAkt phosphorylation [45], only partially suppressed *repo>dEGFR^λ^;dp110^CAAX^* glial overproliferation (Figure 6F), arguing that TORC2 has additional roles. Notably, on their own, *dSin1^−/−^* and *dRictor^−/−^* mutant flies did not show any detectable glial defects (Figure 6G, data not shown). Thus, TORC2 is dispensible for normal glial development, but is necessary for dEGFR^λ^;dp110^CAAX^ glial neoplasia.

TORC1, a complex including Tor and the Raptor regulatory protein, drives cellular growth by stimulating protein synthesis through its effectors S6 kinase and the eIF-4E translation initiation factor [47]. Akt and Erk stimulate TORC1 through phosphorylation and inactivation of the TSC1-TSC2 protein complex, which activates Rheb, and stimulates TORC1 kinase activity. We tested TORC1 function by glial-specific overexpression of dsRNAs for the single *Drosophila* orthologs of Raptor (dRaptor), S6-kinase (dS6K), and eIF4E (deIF4E); these all significantly reduced accumulation of dEGFR^λ^;dp110^CAAX^ mutant glial cells, but only caused mild glial hypoplasia in controls (Figure 6I–K and Figure S10). Co-overexpression of d4EBP, a deIF4E antagonist and dFoxO target gene, also blocked glial neoplasia (Figure 6L). Glial-specific RNAi of dTSC1 enhanced *repo>dEGFR^λ^;dp110^CAAX^* glial overgrowth (Figure 6H). However, overexpression of dTSC1^dsRNA^, dRheb, activated dS6K (dS6K^act^), or deIF4E alone did not produce glial overproliferation, even though these constructs can mimic TORC1 activation (Figure S10, data not shown) [47],[49]. Thus, TORC1 activity is necessary for EGFR-PI3K-driven glial neoplasia, but is not sufficient. Moreover, neither deIF4E nor dS6K^act^ produced glial neoplasia when co-overexpressed with dEGFR^λ^ (data not shown), illustrating that additional dTor-dependent outputs synergize with dEGFR signaling to drive neoplasia.

### Myc and Max Drive EGFR;PI3K Glial Neoplasia via CyclinD-Cdk4

dTor coordinates increased translation, mediated by dS6K and deIF4E, with expression of cell cycle regulators and ribosomal components, through dMyc, the single *Drosophila* ortholog of the Myc bHLH transcription factors [49]. Within developing epithelial tissues, dMyc is required for TORC1-dependent growth and can substitute for dTor activity [49],[50]. Myc protein levels can also be posttranslationally upregulated by EGFR-Ras-Raf signaling [16],[51]. Thus, we suspected that dMyc might mediate signal integration between EGFR-Ras and PI3K.

**Figure 7:**
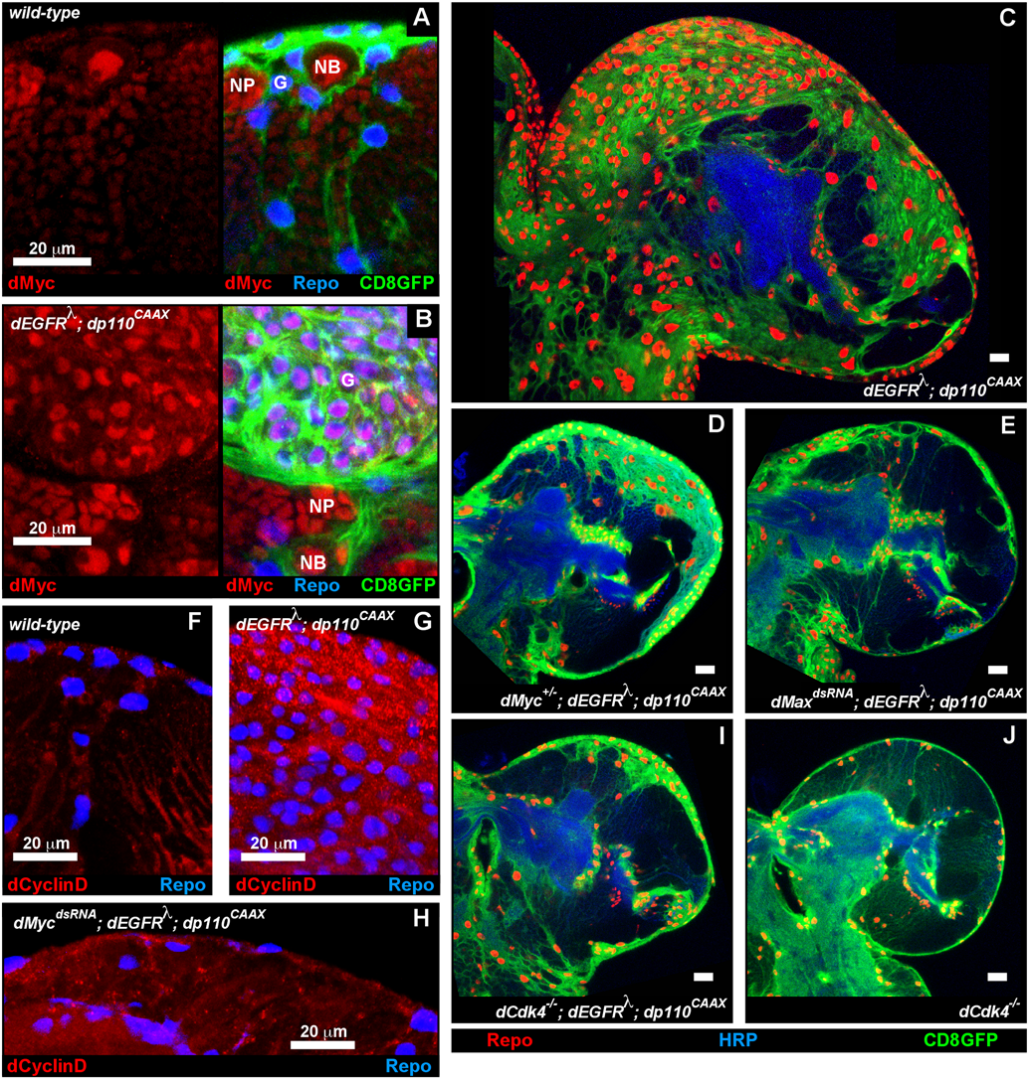
Myc and Max are required to drive EGFR;PI3K glial neoplasia via CyclinD-Cdk4. Frontal sections of 3^rd^ instar larval brains. Anterior up; midline to left. 20 µm scale bars. (A,B) Myc expression (red) in medial anterior regions of wild-type (A) and *repo>dEGFR^λ^;dp110^CAAX^* brains (B). dMyc labeling (red) is shown alone (left) and with overlaid Repo (blue) and CD8GFP (green) glial markers (right). dMyc is highly expressed in neuroblasts (‘NB’) and moderately expressed in ganglion mother cell neuronal progenitors (‘NP’) in both genotypes. Relative to wild-type (A), *repo>dEGFR^λ^;dp110^CAAX^* brains (B) have high levels of dMyc in glia (‘G’), visible as purple nuclei in overlay (B, right panel). 4.5 µm optical projections. (C–E,I,J) 2 µm optical sections midway through larval brain hemispheres from late 3^rd^ instar larvae, displayed at the same scale. Repo (red) labels glial cell nuclei. *repo>CD8GFP* reporter (green) labels glial cell bodies and membranes. HRP (blue) reveals neuropil at high intensity and neuronal cell bodies at low intensity. *dMyc^+/−^; repo>dEGFR^λ^;dp110^CAAX^* (D), *repo>dMax^dsRNA^;dEGFR^λ^;dp110^CAAX^* (E), and *dCdk4^−/−^; repo>dEGFR^λ^;dp110^CAAX^* (I) brains have decreased numbers of glia compared to *repo>dEGFR^λ^;dp110^CAAX^* (C). (J) *dCdk4^−/−^* larval brains have normal gross morphology and glial cell numbers. (F–H) dCyclinD expression (red) overlaid with Repo expression (blue). *repo>dEGFR^λ^;dp110^CAAX^* brains (G) show increased dCyclinD (red) compared to wild-type (F). dCyclinD expression decreases when dMyc is knocked-down in *repo>dMyc^dsRNA^;dEGFR^λ^;dp110^CAAX^* brains (H). 11 µm optical projections. Genotypes: (A) *repo-Gal4 UAS-CD8GFP/+*, (B) and (C) *UAS-dEGFR^λ^ UAS-dp110^CAAX^/+; repo-Gal4 UAS-CD8GFP/+*, (D) *dMyc^P0/+^/UAS-dEGFR^λ^ UAS-dp110^CAAX^; repo-Gal4 UAS-CD8GFP/+*, (E) *UAS-dEGFR^λ^ UAS-dp110^CAAX^/+; UAS-dMax^dsRNA^/+; repo-Gal4 UAS-CD8GFP/+*, (F) *repo-Gal4, UAS-GFP^nls^/+*, (G) *UAS-dEGFR^λ^ UAS-dp110^CAAX^/+; repo-Gal4 UAS-GFP^nls^/+*, (H) *UAS-dEGFR^λ^ UAS-dp110^CAAX^/+; UAS-dMyc^dsRNA^/+; repo-Gal4 UAS-GFP^nls^/+*, (I) *UAS-dEGFR^λ^ UAS-dp110^CAAX^/+; dCdk4^3/k06503^; repo-Gal4 UAS-CD8GFP/+*, (J) *dCdk4^3/k06503^; repo-Gal4 UAS-CD8GFP*.

dEGFR^λ^;dp110^CAAX^ glia showed high levels of nuclear dMyc compared to wild-type glia (Figure 7A and 7B). dMyc was also highly expressed in wild-type neuroblasts (Figure 7A), suggesting that dMyc promotes a proliferative progenitor state [21]. Genetic reduction of *dMyc* with a dsRNA or a single loss-of-function allele strongly suppressed dEGFR^λ^;dp110^CAAX^ glial neoplasia (Figure 7D, data not shown). In fact, some *dMyc^+/−^; repo>dEGFR^λ^;dp110^CAAX^* animals were rescued to viability, indicating that dMyc is an essential rate-limiting output of EGFR-PI3K coactivation.

Myc proteins activate transcription through heterodimerization with the bHLH Max. Max activity was also required for glial neoplasia; a dsRNA for dMax, the single *Drosophila* Max ortholog, strongly suppressed *repo>dEGFR^λ^;dp110^CAAX^* overgrowth (Figure 7E). dMyc-dMax heterodimers promote proliferation by activating expression of multiple cell cycle genes, including dCyclinD and dCdk4 [52]. dCyclinD expression, which is high in *repo>dEGFR^λ^;dp110^CAAX^* glia, was inhibited by a dMyc^dsRNA^ (Figure 7F–H), suggesting that dMyc reduction suppresses glial neoplasia through reduced dCyclinD-dCdk4 activity. To test this we used loss-of-function mutations in dCdk4, which are viable [23]. *dCdk4^−/−^; repo>dEGFR^λ^;dp110^CAAX^* larvae showed near complete absence of excess glia and rescue of glial morphogenesis defects (Figure 7I). Moreover, a dCdk4^dsRNA^ and dCyclinD^dsRNA^ suppressed *repo>dEGFR^λ^;dp110^CAAX^* (data not shown). Thus, dCyclinD-dCdk4 is essential for EGFR-PI3K glial neoplasia, although dCdk4 is not required for development (Figure 7J).

### Myc Overexpression or Rb Loss Synergizes with EGFR

**Figure 8:**
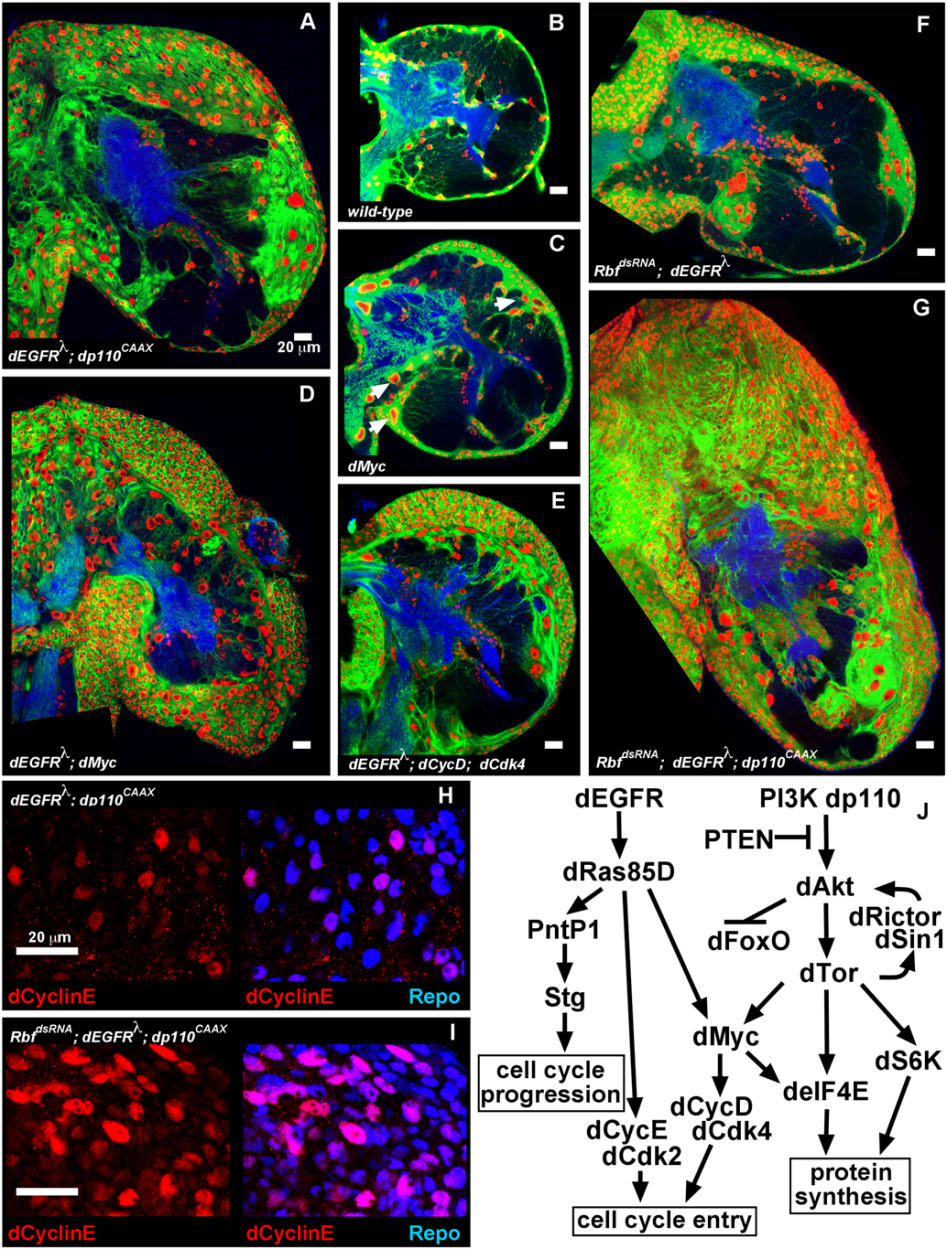
Myc overexpression or Rb loss synergizes with EGFR. (A–G) 2 µm optical sections of larval brain hemispheres from late 3^rd^ instar larvae, displayed at the same scale. 20 µm scale bars. Frontal sections, midway through brains. Anterior up; midline to left. Glial cell nuclei are labeled with Repo (red); glial cell bodies and membranes are labeled with CD8GFP (green) driven by *repo-Gal4*; HRP (blue) reveals neuropil at high intensity and neuronal cell bodies at low intensity. *repo>dMyc* brains (C) contain many glia with enlarged nuclei (arrows). *repo>dEGFR^λ^;dMyc* (D) brains have excess glia (red) throughout the brain, and these glia have very compact cell bodies (green) relative to wild-type glia (B). *repo>dEGFR^λ^;dCyclinD;dCdk4* (E) and *repo>dEGFR^λ^;Rbf1^dsRNA^* (F) brains display increased numbers of glia (red) relative to wild-type (B), but show a less severe glial phenotype and a more normal neuronal compartment (blue) than either *repo>dEGFR^λ^;dp110^CAAX^* (A) or *repo>dEGFR^λ^;dMyc* (D). The combination of *repo>Rbf1^dsRNA^*;*dEGFR^λ^;dp110^CAAX^* (G) enhances neoplasia, leading to a dramatic increase in aberrant glia. (H,I) dCyclinE expression (red) in *repo>dEGFR^λ^;dp110^CAAX^* glia (H) and in *repo>dEGFR^λ^;dp110^CAAX^*;*Rbf1^dsRNA^* glia (I), alone and overlaid with the Repo glial marker (blue, right panels). dCyclinE is more strongly and more broadly expressed in neoplastic glia upon Rbf1 loss, as evidenced by the increased number and intensity of dCyclinE-expressing glial nuclei in (I) compared to (H). Anterior is up, midline to the left. 4.5 µm optical projections; 20 µm scale bars. (J) Pathway diagram of key effectors involved in glial neoplasia initiated by EGFR and PI3K, showing the pathway circuits driving cell cycle entry and progression, and protein translation. Arrows indicate pathway connections, although these connections are not necessarily direct. Genotypes: (A) *UAS-dEGFR^λ^ UAS-dp110^CAAX^/+; repo-Gal4 UAS-CD8GFP/+*, (B) *repo-Gal4 UAS-CD8GFP/+*, (C) *UAS-dMyc/repo-Gal4 UAS-CD8GFP*, (D) *UAS-dEGFR^λ^/+; repo-Gal4 UAS-CD8GFP/UAS-dMyc*, (E) *UAS-dEGFR^λ^/+; UAS-dCyclinD UAS-dCdk4/+; repo-Gal4 UAS-CD8GFP*, (F) *UAS-dEGFR^λ^/+; repo-Gal4 UAS-CD8GFP/UAS-Rbf1^dsRNA^*, (G) *UAS-dEGFR^λ^ UAS-dp110^CAAX^/+; repo-Gal4 UAS-CD8GFP/UAS-Rbf1^dsRNA^*, (H) *UAS-dEGFR^λ^ UAS-dp110^CAAX^/+; repo-Gal4 UAS-CD8GFP/+*, (I) *UAS-dEGFR^λ^ UAS-dp110^CAAX^/+; repo-Gal4 UAS-CD8GFP/UAS-Rbf1^dsRNA^*.

dMyc is necessary for glial neoplasia, but is not sufficient when overexpressed alone (Figure 8C). dMyc-overexpressing glia showed polyploidy (Figure 8C, data not shown), indicating that these cells undergo DNA replication without mitosis, but require additional signals for cell cycle progression. In contrast, co-overexpression of dMyc with dEGFR^λ^ produced a phenotype on par with that of *repo>dEGFR^λ^;dp110^CAAX^* (Figure 8D), indicating that dMyc overexpression can substitute for PI3K activation and promote neoplasia when combined with EGFR signaling.

Given that the dMyc targets dCyclinD-dCdk4 are required for dEGFR^λ^;dp110^CAAX^ neoplasia, we tested whether dCyclinD-dCdk4 overexpression could cooperate with dEGFR^λ^. Additionally, we tested loss of Rbf1, the only known dCdk4 substrate that controls proliferation [23]. *repo>dCyclinD;dCdk4;dEGFR^λ^* and *repo>Rbf1^dsRNA^;dEGFR^λ^* animals showed glial overgrowth, but did not accumulate as many cells as *repo>dEGFR^λ^;dp110^CAAX^* animals (Figure 8E and 8F). Thus, glia likely require additional dp110 or dMyc effectors to undergo full neoplastic proliferation. Other known PI3K and dMyc-dMax target genes that promote proliferation include ribosomal proteins and translation regulators [49],[52], such as eIF4E, which is highly expressed in dEGFR^λ^;dp110^CAAX^ glia and required for neoplasia (Figure 6 and Figure S11).

Our data imply that PI3K, dMyc, and dCyclinD-dCdk4 exist in a linear system, in which Rbf1 inactivation by dCyclinD-dCdk4 is one direct output of dp110^CAAX^ or dMyc. However, in high-grade glioma, Rb loss co-occurs with EGFR and PTEN mutations [2], implying that these mutations cooperate to promote gliomagenesis. To explore interactions between Rb and EGFR-PI3K, we created the triple mutant *repo>Rbf1^dsRNA^;dEGFR^λ^;dp110^CAAX^*. These animals displayed exacerbated glial neoplasia, with a substantial increase in small anaplastic-like glia throughout the CNS (Figure 8G). This synergistic interaction likely derives from derepression of dE2F1 upon Rbf1 loss, and concomitant increased expression of dE2F1 target genes, including Stg and dCyclinE [53]. Increased dCyclinE and Stg expression may accelerate cell cycle progression, perhaps through increased dCdk2 and dCdk1 activity and/or truncated G1 and G2 gap phases caused by constant dCyclinE and Stg protein levels [23]. Consistent with this, we observed increased dCyclinE expression in Rbf1^dsRNA^;dEGFR^λ^;dp110^CAAX^ glia relative to dEGFR^λ^;dp110^CAAX^ glia (Figure 8H and 8I), and co-overexpression of Stg or dCyclinE-dCdk2 with dEGFR^λ^;dp110^CAAX^ synergistically exacerbated glial neoplasia, yielding phenotypes similar to *repo>Rbf^dsRNA^;dEGFR^λ^;dp110^CAAX^* (Figure 5E and Figure S9, data not shown). To assess dCdk2 activity in *repo>Rbf1^dsRNA^;dEGFR^λ^;dp110^CAAX^* brains compared to *repo>dEGFR^λ^;dp110^CAAX^* brains, we stained for phospho-MPM2 (Figure S12), which detects nuclear foci in cells with active dCyclinE-dCdk2 complexes [54]. Phospho-MPM2 foci were present in glia of both genotypes, although *repo>Rbf1^dsRNA^;dEGFR^λ^;dp110^CAAX^* brains appeared to have a higher density of glia with phospho-MPM2 foci (Figure S12), suggesting that expanded expression of dCyclinE results in broader activation of dCdk2. Thus, while PI3K and Rbf1 act in a common genetic pathway linked by dCyclinD-dCdk4, Rbf1 loss nevertheless synergizes with mitogenic stimulation from combined EGFR and PI3K signaling, and this synergy emerges from increased expression of dCyclinE and Stg, rate-limiting regulators of the cell cycle.

## Discussion

We show that constitutive coactivation of EGFR-Ras and PI3K signaling in *Drosophila* glia and glial precursors gives rise to neoplastic, invasive cells that create transplantable tumor-like growths, mimicking human glioma, and mirroring mouse glioma models. This represents a robust organotypic and cell-type specific *Drosophila* cancer model in which malignant cells are created by mutations in the signature genes and pathways thought to be driving forces in a homologous human cancer. This was not necessarily an expected result since fly and human glia show many biological differences despite displaying important similarities [9],[55],[56]. Through genetic analysis of our model, we identified crucial downstream effectors of EGFR and PI3K signaling, many of which are mutated and/or activated in human glioma. These effectors act in a combinatorial network to coordinately stimulate cell cycle entry and progression, block cell cycle exit, and promote inappropriate cellular growth and migration (Figure 8J). Pathways within this network, while interdependent, act synergistically, rather than additively. Thus, *Drosophila* shows evolutionary conservation of oncogene cooperation.

At least four pathway circuits are necessary for glial neoplasia initiated by EGFR and PI3K signaling, including dRas and dMyc circuits, which induce dCyclinE and dCyclinD to drive cell cycle entry, a Pnt circuit, which induces Stg to promote cell cycle progression, and a Tor-eIF4E-S6K pathway, which provides protein translation necessary for proliferation and growth (Figure 8J). When activated individually, these pathways fail to elicit glial neoplasia, implying a requirement for coordinated stimulation of multiple effectors and inactivation of negative regulators. Orthologs for many of the genes within these pathways, such as dRictor, are implicated in human glioma, although specific roles for some, such as ETS transcription factors, have not been defined despite their expression in glioma [2],[57],[58]. While many of these genes are known EGFR and PI3K pathway components, we did not necessarily expect them to be required for EGFR and PI3K dependent glial neoplasia. Indeed, we have tested many other pathway components and outputs, such as Jun kinase, that did not significantly suppress *repo>dEGFR^λ^;dp110^CAAX^* phenotypes upon reduced function (unpublished data).

Coactivation of EGFR and PI3K signaling upregulates dMyc, which is necessary for glial neoplasia. This is consistent with findings that, in flies and mammals, EGFR-Ras, PI3K, and Tor signaling upregulate Myc protein levels [16],[49],[51],[59],[60]. Myc oncogenes are well-known to cooperate with RTK-Ras signaling to drive neoplastic transformation [51], and we demonstrate that this property of Myc is conserved in flies. We also observed sensitivity to reduced Myc gene dosage in our glioma model, which has also been recently documented in a mouse model of PTEN-dependent glioma [61]. *c-myc* is commonly amplified in gliomas [62], implying that Myc is rate limiting, and *c-myc* amplification may be selected for this reason. D-cyclins, established Myc target genes, and Cdk4 are also commonly amplified and/or overexpressed in gliomas [1],[51]. We observed dMyc-dependent dCyclinD overexpression, and a requirement for dCyclinD-dCdk4 in *repo>dEGFR^λ^;dp110^CAAX^* neoplasia, although dCdk4 itself is not required for normal glial proliferation. Together with our analysis of TORC2, this illustrates that oncogenic EGFR-PI3K co-opts effectors that do not control normal glial development. Similarly, *cdk4^−/−^* mutant mice show normal proliferation in many tissues, but are resistant to ErbB-2-driven breast cancers [63],[64]. Our data argue that Cdk4 activity is a key tumor-specific rate-limiting output of EGFR and PI3K signaling in glioma as well.

In contrast to glia, coactivation of EGFR-Ras and PI3K in neuroblasts, which are fly neural stem cells, does not promote unchecked proliferation, despite the fact that neuroblasts express dMyc and are capable of undergoing neoplastic transformation in response to other genetic mutations [21]. Thus, in *Drosophila*, neither a neural stem cell fate nor Myc activity confer competence to undergo EGFR-PI3K neoplastic transformation. Rather, our results suggest that neoplastic cells arise from committed glial progenitors: *dEGFR-dRas85D;dPTEN^−/−^* clones derived from progenitor cells produce large tumors, and anaplastic cells in *repo>dEGFR^λ^;dp110^CAAX^* brains are concentrated in regions enriched for glial progenitors. Notably, regulated developmental signaling through the EGFR pathway promotes proliferation of normal Repo-expressing glial progenitors [27], and our results show that constitutive EGFR and PI3K signaling prolongs this proliferative progenitor state. Further studies of *Drosophila* glial progenitors and glioma-like cells may illuminate the cellular origins of human gliomas, which are thought to arise from progenitor-like glial cells. Moreover, our results argue that cell-type specific factors govern glial neoplasia. One such factor may be Dap, the single p21/p27 ortholog, which is normally expressed in only 5% of all glia (Figure S4). Perhaps glial progenitors do not express Dap, whereas neuronal progenitors do [26], and this underlies susceptibility to transformation by EGFR-Ras and PI3K. Dap is highly regulated in a cell-type specific manner [26], and studies of Dap regulation in glia may further illuminate the genetic origins of glioma, especially given that lack of p21 expression may underlie the tumorigenic response of mammalian glial progenitors to constitutively active EGFR [65].

While EGFR-Ras and PI3K are commonly upregulated in gliomas and experimental models demonstrate that these pathways are required for tumorigenesis, therapies that target EGFR and PI3K signaling have proven disappointing. This discrepancy between clinical and experimental data has many possible explanations. For example, recent studies have demonstrated that EGFR inhibitors are attenuated by particular mutations found in glioma cells, such as PTEN loss or RTK co-amplification [2]. Addressing these and other possibilities remains a challenge that dictates a need for new experimental models. The results presented here establish *Drosophila* as a viable model system for the study of glioma, offering a complex organismal system for rapidly identifying and evaluating therapeutic targets using genetic approaches. Such a system may be especially useful for distinguishing those genetic mutations and pathways that drive tumorigenesis from the large number of genes that show mutations and altered expression in glioblastomas uncovered by recent genomic analyses of patient samples [66],[67]. Our studies have already identified key rate-limiting genes, such as dCyclinE, Stg, and dMyc, and genes only required for abnormal neoplastic glial proliferation, such as dSin1, dRictor, and dCdk4, which may represent important therapeutic targets in human gliomas.

## Materials and Methods

### Fly Stocks, Genetics, and Culture Conditions

Flies were cultured at 25°C. All genotypes were established by standard genetics. To assess larval brain overgrowth phenotypes, embryos were collected for 6–24 hrs, grown for 120–140 hrs, and wandering 3^rd^ instar larvae were selected for dissection.

Stocks were obtained from the Bloomington Stock Center unless otherwise noted. Other than *UAS-PTEN^dsRNA^* lines from Bloomington, all *UAS-dsRNA* lines were obtained from the VDRC stock center [11]. The following stocks were obtained from other investigators: *UAS-dEGFR^λ^* (T. Schubach), *UAS-dEGFR^Elp^, UAS-dEGFR^wild-type^* (N. Baker), *UAS-dPTEN*, *FRT^40A^ dPTEN^2L117^*, *UAS-dFoxO^SA^* (S. Oldham), *UAS-dap* (I. Hariharan), *UAS-dp110^wild-type^*, *UAS-dCycD, UAS-dCdk4, UAS-dMyc, dMyc^4^* (B. Edgar), *UAS-Rbf1* (N. Dyson), *appl-Gal4* (K. Finley), *pros-Gal4* (B. Ohlstein), *wor-Gal4* (C. Doe), *gcm-Gal4* (V. Hartenstein), *dTor^2L7^, dTor^l(2)k17004^* (R. Bodmer), *dRictor^Δ2^* (S. Cohen), and *stg^CB03726^* (A. Spradling)

### UAS-dsRNA Validation

*UAS-dMyc^dsRNA^*, *UAS-TSC1^dsRNA^* lines were validated in prior publications [48]. *UAS-dsRNA* lines were crossed to *actin-Gal4, ey-Gal4*, and *GMR-Gal4* to assess phenotypes. Lines that showed phenotypes inconsistent with known phenotypes for their target genes were excluded from analysis. Gene knock-down in *repo-Gal4* glia was verified with immunohistochemical stains for the following constructs: *UAS-dMyc^dsRNA^*, *UAS-dAkt^dsRNA^*, *UAS-dS6K^dsRNA^, UAS-eIF4E^dsRNA^, UAS-Rbf1^dsRNA^*, and *UAS-pnt^dsRNA^* (Figure S11).

### Abdominal Transplants

Larval brains were dissected into sterile PBS, washed, and cut into fragments. Abdominal incisions were made in virgin female hosts and single brain fragments were inserted. Hosts were cultured for 1–6 weeks, dissected and fixed in 4% paraformaldehyde, incubated in 10% sucrose and embedded in O.C.T. Thick 50 µm sections were stained as described below.

### Clonal Analysis

For hs-FLP clones, genotypes are indicated in figure legends. Flies were initially grown at 18°C or 20°C to minimize spontaneous clones, which occurred at a low frequency during late larval-pupal stages. 3^rd^ instar larvae, 0–48 hr pupae, or 0–2 day old young adults were treated with heat shock to induce clones and subsequently cultured at 25°C for 1–4 weeks. For ey-FLP clones, flies were cultured at 25°C continuously.

### Immunohistochemistry and Imaging

Larval tissue was fixed for 30–50 minutes in 1×PBS 4% paraformaldehyde. Adult brains were fixed for 1–2 hr in 1×PBS 4% paraformaldehyde or in PLP with 2% paraformaldehyde. For BrdU labeling, larvae were cultured in food with 1 mg/ml BrdU for 4–6 hrs, and fixed larval brains were treated with 2 N HCl for 30 minutes followed by DNase for 1 hr. Stains were performed in 1×PBS 10% BSA with 0.3% Triton-X100 for larval brains and 0.5% Triton-X100 for adult samples.

The following antibodies were obtained from the Developmental Studies Hybridoma Bank and diluted 1∶5–1∶10: 8D12 anti-Repo, anti-dMMP1, anti-dCyclinB, anti-Elav, and 40-1a anti-lacZ. Larval and/or adult brains were also stained with rabbit anti-Repo (G. Technau, 1∶500), rat anti-dCyclinE (H. Richardson, 1∶100), anti-BrdU (BD, 1∶100), rat anti-Miranda (C. Doe, 1∶100), mouse anti-diphospho-Erk (Sigma, 1∶200), mouse anti-Rbf1 (N. Dyson, 1∶5), mouse anti-Dap (I. Hariharan, 1∶10), rabbit anti-PntP1 (J. Skeath, 1∶500), rabbit anti-eIF4E (P. Lasko, 1∶100), rabbit anti-dMyc (D. Stein, 1∶1000), and anti-phospho-MPM2 (Upstate Biotechnology, 1∶200). Anti-HRP-Cy5 and anti-HRP-Cy3 (Jackson Labs) were used at 1∶250–1∶500. Secondary antibodies were conjugated to Cy3 (Jackson Labs) or Alexa-488 or Alexa-647 (Molecular Probes). Actin was visualized with Rhodamine-labeled phalloidin (Invitrogen).

Brains were imaged as whole mounts on a Zeiss LSM 510 confocal system. Images were analyzed in Zeiss LSM Image Browser and processed in Photoshop CS3. For experiments in which protein levels were compared between genotypes, all sample preparation, histochemistry, imaging, and image processing was performed in parallel in the same manner.

## Supporting Information

Figure S1 Diagram of *Drosophila* EGFR mutant forms. Proteins are shown as horizontal bars along which functional domains are indicated, and the locations of alterations in mutant forms are noted. Wild-type human EGFR and *Drosophila* EGFR (dEGFR) show extensive conservation, with 55% homology in the kinase domain and 41% homology in the ligand-binding portion of the extracellular domain, which includes extensive cysteine repeats. The signal peptide is labeled in yellow. Following the signal peptide, the entire cytoplasmic domain is replaced with the lambda dimerization domain in dEGFR^λ^, which causes constitutive activation. Both human EGFR and dEGFR show extensive cysteine repeats in the extracellular domain, indicated in blue. The tyrosine kinase domain is labeled in magenta. The Elp mutant form of dEGFR contains an A887T substitution in the N-lobe of the kinase domain, which causes constitutive activation.
(0.23 MB TIF)

Figure S2 Coactivation of Ras-Raf and PI3K in *Drosophila* glia causes neoplasia. 2 µm optical sections of representative larval brain hemispheres from wandering 3rd instar larvae (A–H) and an early 2nd instar larval brain (I), all displayed at the same scale. 20 µm scale bars. Frontal sections; midway through brains. Anterior up; midline to left. Glial cell nuclei are labeled with Repo (red). CD8GFP (green), driven by the *repo-Gal4* driver, labels glial cell bodies and membranes. An anti-HRP counterstain (blue) reveals neuropil (neuronal fiber tracts) at high intensity and some cell bodies of neurons and neuronal precursors at low intensity, and this varies slightly according to exact plane of section and mutant phenotype. *repo>dEGFRλ;dp110CAAX* (A), *repo>dRas85DV12;dPTENdsRNA* (B), and *repo>dRafgof;dp110CAAX* (C) brains show increased numbers of glia relative to wild-type (D), *repo>dRas85DV12* alone (E), or *repo>dPTENdsRNA* alone (F). Compared to wild-type (D), *repo>dEGFRwild-type* (H) brains show reduced neurons (HRP, low intensity blue), which renders glia more densely packed and the entire brain smaller than normal. However, *repo>dEGFRwild-type* brains (H) do not show a substantial increase the number of glia compared to wild-type (D). Brains of *repo>dEGFRElp* 2nd instar larvae (I) show substantial neuron loss, brain malformation, and excess glia for that stage of development. Genotypes: (A) *UAS-dEGFRλ UAS-dp110CAAX/+; repo-Gal4 UAS-CD8GFP/+* (B) *UAS-CD8GFP/+; repo-Gal4/UAS-dRas85DV12 UAS-dPTENdsRNA* (C) *UAS-dp110CAAX; repo-Gal4 UAS-CD8GFP/UAS-dRafgof* (D) *repo-Gal4 UAS-CD8GFP/+* (E) *repo-Gal4 UAS-CD8GFP/UAS-dRas85DV12* (F) *UAS-CD8GFP/+; repo-Gal4/UAS-dPTENdsRNA* (G) *UAS-dEGFRλ/+; repo-Gal4 UAS-CD8GFP/+* (H) *UAS-CD8GFP/+; repo-Gal4/UAS-dEGFRwild-type* (I) *UAS-CD8GFP/+; repo-Gal4/UAS-dEGFRElp*.
(10.31 MB TIF)

Figure S3 dMMP1 expression in wild-type and dEGFR^λ^;dp110^CAAX^ glia. (A,B) 3rd instar larval brains. Frontal sections, showing medial regions enriched for proliferating glia. Anterior up; midline to left. 4.5 µm optical projections, matched in scale. 20 µm scale bars. Expression of the active form of dMMP1 (red) in wild-type (A) and *repo>dEGFRλ;dp110CAAX* brains (B), shown alone (left panels) and overlaid with an HRP (blue) neuronal label and a CD8GFP (green) glial label (right panels). In wild-type brains, glia (‘G’) rarely express active dMMP1, although some glia on the surface of the brain show low levels of dMMP1 staining (arrow). In *repo>dEGFRλ;dp110CAAX* brains (B), some neoplastic glia (‘G’) express active dMMP1 (red in right panel, yellow in overlay), which is largely membrane-localized in individual cells (arrow). Genotypes: (A) *UAS-CD8GFP/+; repo-Gal4/+* (B) *UAS-dEGFRλ UAS-dp110CAAX/+; UAS-CD8GFP/+; repo-Gal4/+*.
(3.93 MB TIF)

Figure S4 Glial-specific Rbf1 knock-down or overexpression of G1 Cyclin-Cdks, Dap, and Rbf1 affects glial proliferation. (A–F) 2 µm optical sections of larval brain hemispheres from wandering 3rd instar larvae displayed at the same scale. 20 µm scale bars. Frontal sections; midway through brains. Anterior up; midline to left. Glial cell nuclei are labeled with Repo (red). Glial cell bodies and membranes are labeled with CD8GFP (green) driven by *repo-Gal4*. HRP counter-staining (blue) reveals neuropil at high intensity and some cell bodies of neurons and neuronal precursors at low intensity. Rbf1 knock-down in *repo>Rbf1dsRNA* (B) does not significantly alter glial cell numbers (red nuclei) compared to wild-type (A). Ectopic expression of dCyclinE-dCdk2 (C) or dCyclinD-dCdk4 in *repo-Gal4*-glia promotes an approximate doubling of glial cells numbers (red nuclei), even when combined with Rbf1^dsRNA^ (D). Continuous expression of Dap (E) or Rbf1 (F) in otherwise wild-type *repo-Gal4*-glia substantially reduces glial cell numbers, showing that *repo-Gal4* glia normally undergo proliferation controlled by dCyclinE-dCdk2 and Rbf1-E2F1. Genotypes: (A) *UAS-CD8GFP/+; repo-Gal4/+* (B) *UAS-CD8GFP/+; repo-Gal4/UAS-Rbf1dsRNA* (C) *UAS-CD8GFP/+; repo-Gal4/UAS-dCyclinE UAS-dCdk2* (D) *UAS-CD8GFP/UAS-dCyclinD UAS-dCdk4; repo-Gal4/UAS-Rbf1dsRNA* (E) *UAS-dap/+; UAS-CD8GFP/+; repo-Gal4/+* (F) *UAS-CD8GFP/+; repo-Gal4/UAS-Rbf1*.
(8.27 MB TIF)

Figure S5 Dap and Rbf1 expression in wild-type and dEGFR^λ^;dp110^CAAX^ glia. 3rd instar larval brains. Frontal sections, showing superficial dorsal regions enriched for Dap- and Rbf1-expressing cells. Anterior up; midline to left. 7 µm optical projections, all matched in scale. 20 µm scale bars. (A,B) Dap expression (red) in wild-type (A) and *repo>dEGFRλ;dp110CAAX* brains (B), shown alone (left panels) and overlaid with Repo (blue) and *repo>CD8GFP* (green) glial markers (right panels). In both genotypes, Dap is primarily expressed in neuroblasts (‘NB’) and ganglion mother cell neuronal precursors (‘NP’), rarely in glia (‘G’), and almost never in neurons (‘N’). In *repo>dEGFRλ;dp110CAAX* brains, Dap is rarely expressed in glia as seen by the lack of substantial overlap between Dap expression and glial markers (B, right panel). White arrows denote rare Dap-positive glia in both wild-type and *repo>dEGFRλ;dp110CAAX* brains; these glia show lower levels of Dap protein than neighboring neuroblasts and neuronal precursors. Dap-positive glia were counted in 3 *repo>dEGFRλ;dp110CAAX* brains and an average of 5% of all glia expressed Dap (87 glia expressed Dap out of 1738 total glia counted). (C–D) Rbf1 expression (red) in wild-type (E) and *repo>dEGFRλ;dp110CAAX* brains (F), shown alone (left) and overlaid (right) with the CD8GFP (green) glial marker and HRP (blue) neuronal marker. In both genotypes, Rbf1 is highly expressed in glia (‘G’) and neuroblasts (‘NB’), which were identified by their characteristic positions and large cell bodies. Neurons (‘N’) show lower expression. Genotypes: (A,C) *UAS-CD8GFP/+; repo-Gal4/+* (B,D) *UAS-dEGFRλ UAS-dp110CAAX/+; UAS-CD8GFP/+; repo-Gal4/+*.
(7.96 MB TIF)

Figure S6 dEGFR^λ^; dp110^CAAX^ glia do not express neuronal and neuroblast markers. Late 3rd instar larval brains. Medial anterior regions shown. Anterior up; midline to left. 2 µm optical sections. CD8GFP (green), driven by the *repo-Gal4* driver, labels glial cell bodies and membranes. An HRP counter-stain (blue) reveals neuropil at high intensity and cell bodies of neurons and some neuronal precursors at low intensity. (A,B) Repo expression (red) in glial cell nuclei in wild-type (A) and *repo>dEGFRλ;dp110CAAX* brains (B). In wild-type brains, neuronal fibers and neurons (blue) are enveloped by glial processes (green) to give the brain a honeycombed appearance. In *repo>dEGFRλ;dp110CAAX* brains, neurons (‘N’) lack GFP or Repo expression, in contrast to glia (‘G’). (C–D) Elav expression (red) in wild-type (C) and *repo>dEGFRλ;dp110CAAX* brains (D), shown alone (left panels) and overlaid (right panels) with the CD8GFP (green) glial marker and HRP (blue) neuronal marker. In both genotypes, Elav is highly expressed in neurons (‘N’) but absent from glia (green cell bodies indicated by white arrows). (E–F) Miranda expression (red) in wild-type (E) and *repo>dEGFRλ;dp110CAAX* (F) brains, shown alone (left panels) and overlaid (right panels) with CD8GFP (green) glial marker and HRP (blue) neuronal marker. In both genotypes, Miranda is highly expressed in neuroblasts (‘NB’), which are identified by their characteristic positions and large cell bodies, but is absent from glia (green cell bodies, indicated by white arrows). Genotypes: (A,C,E) *UAS-CD8GFP/+; repo-Gal4/+* (B,D,F) *UAS-dEGFRλ UAS-dp110CAAX/+; UAS-CD8GFP/+; repo-Gal4/+*.
(9.24 MB TIF)

Figure S7 dEGFR^λ^ and dp110^CAAX^ do not induce neoplasia from neurons, neuroblasts, or certain glia. Late 3rd instar larval brain hemispheres. Medial anterior regions shown. Anterior up; midline to left. 3.5 µm optical projections. Repo expression (red) in glial cell nuclei in all genotypes. An HRP counter-stain (blue) reveals neuropil at high intensity and cell bodies of neurons and some neuronal precursors at low intensity, and this varies slightly according to variance in exact section plane. (A–D) Wild-type (A), dEGFR^λ^; dp110^CAAX^ overexpressed using the *1407-Gal4* neuroblast driver (B), dEGFR^λ^; dp110^CAAX^ overexpressed using the *pros-Gal4* neural driver (C), and dEGFR^λ^;dp110^CAAX^ overexpressed using the *gcm-Gal4* embryonic glial driver (D). In all cases, dEGFR^λ^;dp110^CAAX^ overexpression did not induce neoplastic overgrowth of glial or neuronal cell types, as determined by brain size and cell-type specific stains. (E,F) Cytoplasmic GFP (green), driven by the *Nrv2-Gal4* driver, labels cell bodies and cytoplasmic processes, as seen in wild-type (E). dEGFR^λ^;dp110^CAAX^ overexpressed using the *Nrv2-Gal4* glial driver (B). *Nrv2-Gal4* is expressed by post-mitotic cortex glia, which proliferate somewhat in response to dEGFR^λ^;dp110^CAAX^ to cause slight brain enlargement, but do not become neoplastic like *repo>dEGFRλ;dp110CAAX* glia. Genotypes: (A) *+/CyO* (B) *UAS-dEGFRλ UAS-dp110CAAX/+; 1407-Gal4/UAS-Gal4* (C) *UAS-dEGFRλ UAS-dp110CAAX/+; UAS-Gal4/+; pros-Gal4/+* (D) *UAS-dEGFRλ UAS-dp110CAAX/+; gcm-Gal4/+* (E) *Nrv2-Gal4 UAS-GFP/+; Nrv2-Gal4 UAS-GFP/+* (F) *UAS-dEGFRλ UAS-dp110CAAX/+; Nrv2-Gal4 UAS-GFP/UAS-Gal4; Nrv2-Gal4 UAS-GFP/+*.
(7.96 MB TIF)

Figure S8 Coactivation of EGFR and PI3K in glial progenitors creates invasive neoplastic glia.(A–E) FLP/FRT clones in adult brains derived from a population of ey-FLP and *repo-Gal4*-expressing cells. CD8GFP (green) marks cell bodies and membranes of glial clones derived by FLP/FRT mitotic recombination (see text). Repo (red) marks all glial cell nuclei, in both clones and surrounding normal tissue. 8.5 µm confocal optical sections through brains of similarly aged adults, all matched to scale. 20 µm scale bars. Each panel shows half brains, including a whole optic lobe and adjacent central brain. *dEGFRElp* (C) and *dPTEN−/−* (J) clones are composed of 2–5-fold more cells than wild-type controls (H). In contrast, *dEGFRλ;dPTEN−/−* (D) and *dEGFRElp;PTEN−/−* double mutant clones form large tumors visible in adult brains. As with hs-FLP clones, *dEGFRλ;dPTEN−/−* and *dEGFRElp;dPTEN−/−* clones are less cellular and more invasive than *dRas85DV12;PTEN−/−* clones (see Figure 4). Genotypes: (A) *ey-flp/+; FRT40A tubGal80/FRT,40A; repo-Gal4 UAS-CD8GFP/+* (B) *ey-flp/+; FRT40A tubGal80/FRT40A PTEN2L117; repo-Gal4 UAS-CD8GFP/+* (C) *ey-flp/+; FRTG13 tubGal80/FRTG13 UAS-CD8GFP; repo-Gal4/UAS-dEGFRElp* (D) *ey-flp/UAS- dEGFRλ; FRT40A tubGal80/FRT40A PTEN2L117; repo-Gal4 UAS-CD8GFP/UAS- dEGFRλ* (E) *ey-flp/+; FRT40A tubGal80/FRT40A PTEN2L117; repo-Gal4 UAS-CD8GFP/UAS-dEGFRElp*.
(7.84 MB TIF)

Figure S9 Stg overexpression exacerbates EGFR-PI3K glial neoplasia. 2 µm optical sections of larval brain hemispheres from late 3rd instar larvae, displayed at the same scale. 20 µm scale bars. Frontal sections, midway through brains. Anterior up; midline to left. Glial cell nuclei are labeled with Repo (red); glial cell bodies and membranes are labeled with CD8GFP (green) driven by *repo-Gal4*. An HRP counter-stain (blue) reveals neuropil at high intensity and neuronal cell bodies at low intensity. HRP stains varied between the two samples according to effects of mutant glia and slight variance in section plane. Stg co-overexpression with dEGFR^λ^;dp110^CAAX^ (B) enhances neoplasia compared to dEGFR^λ^;dp110^CAAX^ (A), leading to a dramatic increase in aberrant glia and yielding a phenotype similar to that of *repo>Rbf1dsRNA;dEGFRλ;dp110CAAX* (see Figure 8G). Genotypes: (A) *UAS-dEGFRλ UAS-dp110CAAX/+; repo-Gal4 UAS-CD8GFP/+* (B) *UAS-dEGFRλ UAS-dp110CAAX/+; UAS-stg/+; repo-Gal4 UAS-CD8GFP/+*.
(6.27 MB TIF)

Figure S10 Knock-down and/or overexpression of dTor and dTor effectors. 2 µm optical sections of larval brain hemispheres from wandering 3rd instar larvae, approximately 130hr AED, all displayed at the same scale. 20 µm scale bars. Anterior up; midline to the left. Frontal sections; midway through brains. Repo (red) marks glial cell nuclei; CD8GFP (green), driven by *repo-Gal4*, labels glial cell bodies and membranes. An HRP-counter stain (blue) reveals neuropil (neuronal fiber tracts) at high intensity and some cell bodies of neurons and neuronal precursors at low intensity, and this varies slightly according to exact plane of section, brain orientation, and mutant phenotype. *repo>dAktdsRNA* brains (B) are smaller and have fewer glia than wild-type controls (A). All other genotypes have relatively normal sized brains. By gross examination, *repo>TorTED* (C), *repo>dRaptordsRNA* (E), *repo>deIF4EdsRNA* (F) and *repo>dS6KdsRNA* (G) display an estimated 10–20% reduction in glial cell numbers. *repo>dTSC1dsRNA* (D), *repo>Rheb* (H) and *repo>deIF4E* (I) brains contain normal numbers of glia. Genotypes: (A) *repo-Gal4 UAS-CD8GFP/+* (B) *UAS-CD8GFP/+; repo-Gal4/UAS-dAktdsRNA* (C) *UAS-TorTED/UAS-CD8GFP; repo-Gal4/+* (D) *UAS-CD8GFP/+; repo-Gal4/UAS-dTSC1dsRNA* (E) *UAS-dRaptordsRNA/UAS-CD8GFP; repo-Gal4/+* (F) *UAS-CD8GFP/+; repo-Gal4/UAS-deIF4EdsRNA* (G) *UAS-dS6KdsRNA; UAS-CD8GFP/+; repo-Gal4/+* (H) *UAS-CD8GFP/+; repo-Gal4/UAS-Rheb* (I) *UAS-CD8GFP//UAS-deIF4E; repo-Gal4/+*.
(8.83 MB TIF)

Figure S11 Validation of dsRNA constructs. 2 µm optical sections of larval brain hemispheres from wandering 3rd instar larvae. Frontal sections. Anterior up, midline to the left. Each individual staining pattern is shown alone (left panels) and with overlaid glial or neuronal markers (right panels). Repo (blue) in (A–J) marks glial cell nuclei. Glial cell bodies and membranes are labeled with CD8GFP (green) driven by *repo-Gal4*. In (K,L) an HRP counter-stain (blue) reveals neurons. Red marks histochemical stains for each indicated protein in *repo>dEGFRλ;dp110CAAX* (A,C,E,G,I,K) and *repo>dEGFRλ;dp110CAAX* with each indicated dsRNA construct (B,D,F,H,J,L). Each dsRNA construct was expressed with *repo-Gal4* to yield glial-specific knock-down, which left protein expression in surrounding neuronal tissue intact. For the nuclear protein PntP1, knock-down was verified by the lack of glial-specific staining in the presence of the *pntdsRNA* (B), although PntP1 is present within neighboring neurons (‘N’). For dAkt, deIF4E, and dS6K, gene knock-down was confirmed using antibodies for total protein. Glial-specific reduction in gene expression by *dAktdsRNA* and *deIF4EdsRNA* is highlighted by white outlines in (D) and (F). dAkt protein is higher in neuronal tissue (‘N’) in both *repo>dEGFRλ;dp110CAAX* (C) and *repo>dAktdsRNA;dEGFRλ;dp110CAAX* (D). deIF4E is high in dEGFR^λ^;dp110^CAAX^ glia (E) and low in neurons (E,F). *repo>deIF4EdsRNA;dEGFRλ;dp110CAAX* brains (F) have reduced glial eIF4E. Reduced glial dS6K protein, which is diffusely cytoplasmic, is observed in *repo>dS6KdsRNA;dEGFRλ;dp110CAAX* brains (H) compared to *repo>dEGFRλ;dp110CAAX* (G). Reduced glial dMyc expression in *repo>dMycdsRNA;dEGFRλ;dp110CAAX* brains (J) is noted by white outlines, and the absence of purple nuclei in overlay (J, right panel), relative to *repo>dEGFRλ;dp110CAAX* (I, right panel). dMyc is highly expressed in neuroblasts (‘NB’) in both samples (I, J), which do not express the *dMycdsRNA*. Glial Rbf1 protein is absent in *repo>dRbf1dsRNA;dEGFRλ;dp110CAAX* brains (L) in contrast to neighboring neurons (‘N’) in ,*repo>dEGFRλ;dp110CAAX* brains (K). Genotypes: (A,C,E,G,I,K) *UAS-dEGFR, λ UAS-dp110,CAAX/+; UAS-CD8GFP/+; repo-Gal4/+* (B) *UAS-dEGFRλ UAS-dp110CAAX/+; repo-Gal4 UAS-CD8GFP/UAS-pntdsRNA* (D) *UAS-dEGFRλ UAS-dp110CAAX/+; repo-Gal4 UAS-CD8GFP/UAS-dAktdsRNA* (F) *UAS-dEGFRλ UAS-dp110CAAX/+; repo-Gal4 UAS-CD8GFP/UAS-deIF4EdsRNA* (H) *UAS-dEGFRλ UAS-dp110CAAX/UAS-dS6KdsRNA; repo-Gal4 UAS-CD8GFP/+* (J) *UAS-dEGFRλ UAS-dp110CAAX/+; UAS-dMycdsRNA/+; repo-Gal4 UAS-CD8GFP/+* (L) *UAS-dEGFRλ UAS-dp110CAAX/+; repo-Gal4 UAS-CD8GFP/UAS-Rbf1dsRNA*.
(9.04 MB TIF)

Figure S12 Phospho-MPM2 reveals increased S-phase and M-phase glia in *repo>dRbf1dsRNA;dEGFRλ;dp110CAAX* brains. (A–C) phospho-MPM2 expression (red) in wild-type brains (A), *repo>dEGFRλ;dp110CAAX* brains (B), and *repo>dRbf1dsRNA;dEGFRλ;dp110CAAX* brains (C). 20 µm scale bars. Anterior up, midline to the left. 6 µm optical projections showing representative superficial dorsal regions enriched for mitotic glia. Phospho-MPM2 shown alone (left panels), overlaid with the Repo (blue, middle panels), and CD8GFP (green, right panels) glial markers. Phospho-MPM2 nuclear foci are present in S-phase cells (arrows note examples). S-phase glia are clearly visible in the middle panel as purple cells with MPM2 foci, which appear enriched in *repo>dRbf1dsRNA;dEGFRλ;dp110CAAX* brains (C) relative to *repo>dEGFRλ;dp110CAAX* brains (B). In all genotypes, high levels of phospho-MPM2 is also expressed in mitotic cells (asterisks note examples). Mitotic glia showed low levels of Repo (middle panels), but are clearly GFP-positive (right panels), suggesting that Repo protein expression is reduced upon mitosis in glia. *repo>dRbf1dsRNA;dEGFRλ;dp110CAAX* brains (C) also show increased density of phospho-MPM2-positive mitotic glia compared to *repo>dEGFRλ;dp110CAAX* brains (B). In wild-type (A), the majority of phospho-MPM2-postive cells are not glial and are neuroblasts or neuronal precursors, as revealed by the lack of overlap between phospho-MPM2 staining (middle panel) and the Repo and CD8GFP markers. Genotypes: (A) *repo-Gal4 UAS-CD8GFP/+* (B) *UAS-dEGFRλ UAS-dp110CAAX/+; UAS-CD8GFP/+; repo-Gal4/+* (C) *UAS-dEGFRλ UAS-dp110CAAX/+; repo-Gal4 UAS-CD8GFP/UAS-Rbf1dsRNA*.
(8.99 MB TIF)

Table S1 Overexpression of EGFR-Ras and PI3K-Akt pathways in *Drosophila* glia.
(0.08 MB PDF)

Table S2 Coactivation of EGFR and PI3K does not elicit neoplasia in all neural cell types.
(0.08 MB PDF)

Table S3 Genetic analysis of Akt, Tor, Myc, and CyclinD-Cdk4 pathways in neoplastic and normal glia.
(0.10 MB PDF)

Video S1 An animated 7.5 µm thick confocal z-stack of dEGFR^λ^; dp110^CAAX^ tumor cells derived from transplanted larval glia, pictured in Figure 3D. The depth of each frame is noted in the upper left-hand corner. 20 µm scale bar for x/y-axis. Transplanted mutant glia are labeled with membrane bound CD8GFP (green) and the Repo nuclear protein (blue). Actin staining (red, phalloidin) reveals abdominal anatomy of the host. Asterisks indicate trachea embedded in the pictured dEGFR^λ^;dp110^CAAX^ tumor, visible as hollow actin-positive (red) tubules running through tissues. Genotypes: Hosts were *w1118* virgin females. Transplanted glia were *UAS-dEGFRλ UAS-dp110CAAX/+; UAS-CD8GFP/+; repo-Gal4/+*.
(1.54 MB MOV)

Video S2 An animated 27 µm thick confocal z-stack of dEGFR^λ^; dp110^CAAX^ tumor cells derived from transplanted larval glia, pictured in Figure 3F. The depth of each frame is noted in the upper left-hand corner. 20 µm scale bar for x/y-axis. Transplanted mutant glia are labeled with membrane bound CD8GFP (green) and the Repo nuclear protein (blue). Actin staining (red, phalloidin) reveals abdominal anatomy of the host. Arrowheads in indicate dEGFR^λ^;dp110^CAAX^ glial cells invading an ovary, distinguished by its characteristic actin staining (bright red). An asterisk indicates trachea embedded in this dEGFR^λ^;dp110^CAAX^ tumor, visible as a hollow actin-positive (red) tubule running through the tissue. Genotypes: Hosts were *w1118* virgin females. Transplanted glia were *UAS-dEGFRλ UAS-dp110CAAX/+; UAS-CD8GFP/+; repo-Gal4/+*.
(1.16 MB MOV)

Video S3 An animated 88 µm thick confocal z-stack of a hs-FLP/FRT clone of *dPTEN−/−;dRas85DV12* cells pictured in Figure 4E. The depth of each frame is noted in the upper left-hand corner. 20 µm scale bar for x/y-axis. Approximately half of the affected brain is displayed, with the midline to the left. The *dPTEN−/−;dRas85DV12* mutant cells are labeled with membrane-bound CD8GFP (green) and penetrate through the entire depth of the brain, some of which is visible as red background. These *dPTEN−/−;dRas85DV12* cells appear to originate in the optic lobe (right) and extend variable tendrils into the central brain (middle, left). Genotype: *hs-flp1/+; FRT40A tubGal80/FRT40A PTEN2L117; repo-Gal4 UAS-CD8GFP/UAS-dRas85DV12*.
(1.06 MB MOV)

Video S4 An animated 16 µm thick confocal z-stack of a hs-FLP/FRT clone of wild-type cells pictured in Figure 4A. The depth of each frame is noted in the upper left-hand corner. 20 µm scale bar for x/y-axis. Midline to the left. This clone of normal glia is labeled with membrane-bound CD8GFP (green) and shows cells in a cell-body rich region of the central brain adjacent to the optic lobe. The clone is composed of 2 cells with extensive cytoplasmic projections. Genotype: *hs-flp1/+;FRTG13 tubGal80/FRTG13 UAS-CD8GFP; repo-Gal4/+*.
(0.24 MB MOV)
