# Supplementary figures and images for: A Drosophila Model for EGFR-Ras and PI3K-Dependent Human Glioma
Source: PLoS Genet. 2009 Feb 13;5(2):e1000374. doi: 10.1371/journal.pgen.1000374 (PMC2636203; doi:10.1371/journal.pgen.1000374)

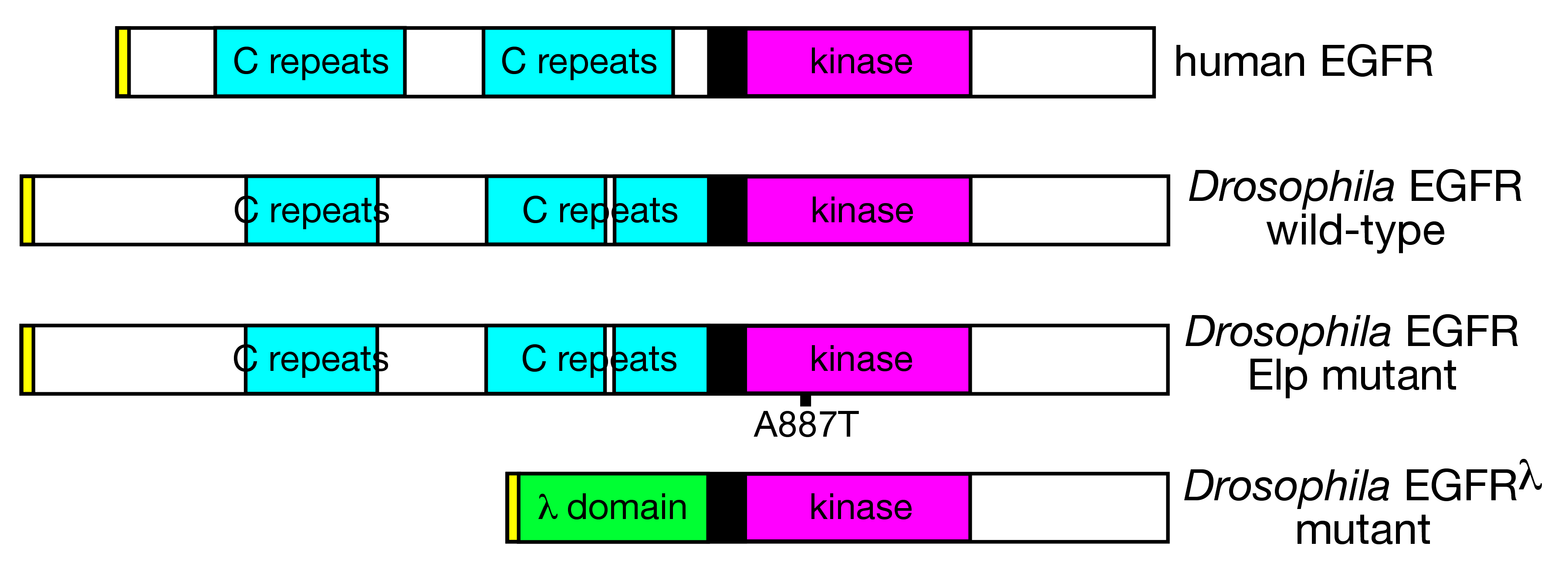

Supplement: Figure S1 — Diagram of Drosophila EGFR mutant forms. Proteins are shown as horizontal bars along which functional domains are indicated, and the locations of alterations in mutant forms are noted. Wild-type human EGFR and Drosophila EGFR (dEGFR) show extensive conservation, with 55% homology in the kinase domain and 41% homology in the ligand-binding portion of the extracellular domain, which includes extensive cysteine repeats. The signal peptide is labeled in yellow. Following the signal peptide, the entire cytoplasmic domain is replaced with the lambda dimerization domain in dEGFRλ, which causes constitutive activation. Both human EGFR and dEGFR show extensive cysteine repeats in the extracellular domain, indicated in blue. The tyrosine kinase domain is labeled in magenta. The Elp mutant form of dEGFR contains an A887T substitution in the N-lobe of the kinase domain, which causes constitutive activation. (0.23 MB TIF) [file pgen.1000374.s001.tif]

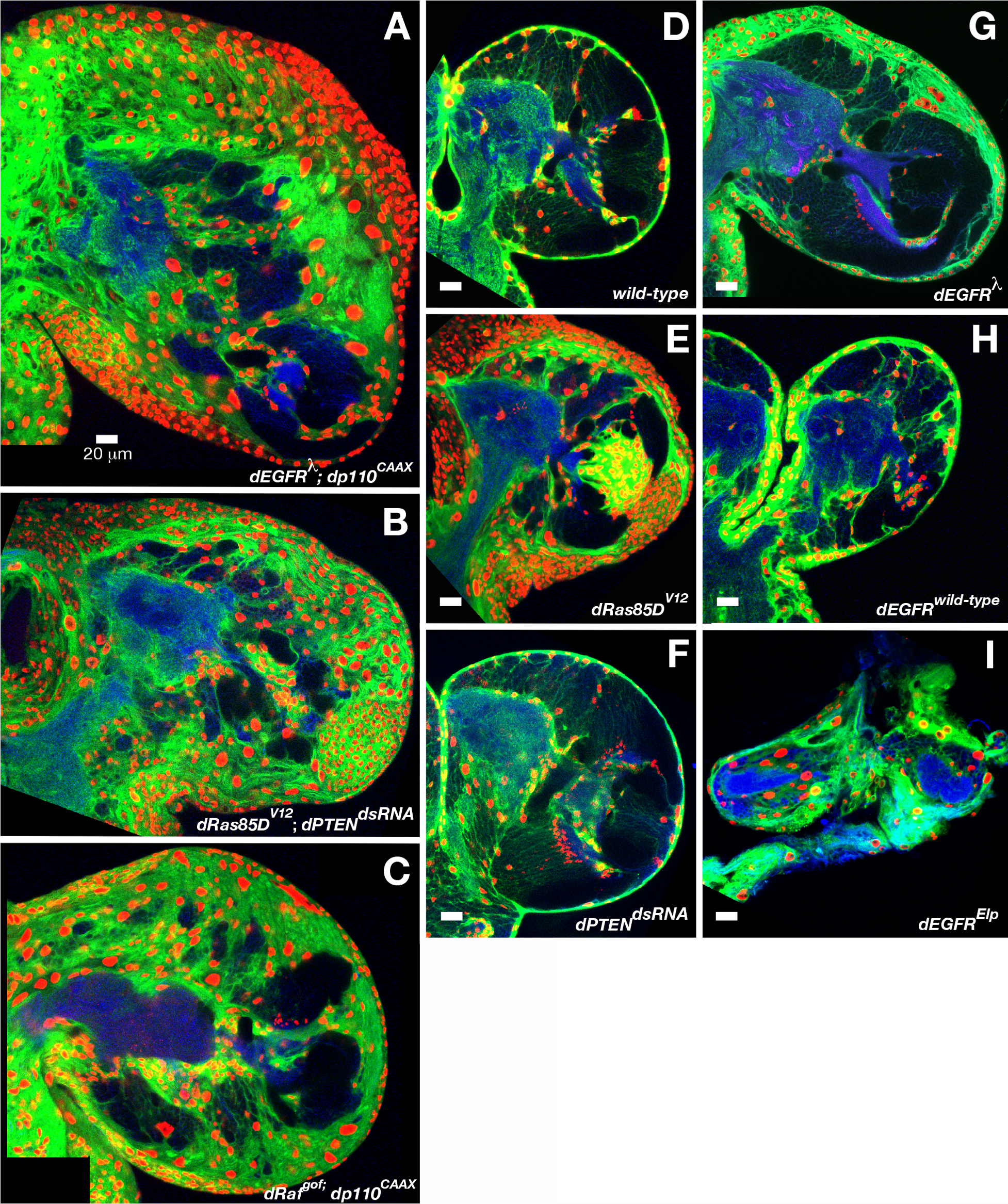

Supplement: Figure S2 — Coactivation of Ras-Raf and PI3K in Drosophila glia causes neoplasia. 2 µm optical sections of representative larval brain hemispheres from wandering 3rd instar larvae (A–H) and an early 2nd instar larval brain (I), all displayed at the same scale. 20 µm scale bars. Frontal sections; midway through brains. Anterior up; midline to left. Glial cell nuclei are labeled with Repo (red). CD8GFP (green), driven by the repo-Gal4 driver, labels glial cell bodies and membranes. An anti-HRP counterstain (blue) reveals neuropil (neuronal fiber tracts) at high intensity and some cell bodies of neurons and neuronal precursors at low intensity, and this varies slightly according to exact plane of section and mutant phenotype. repo>dEGFRλ;dp110CAAX (A), repo>dRas85DV12;dPTENdsRNA (B), and repo>dRafgof;dp110CAAX (C) brains show increased numbers of glia relative to wild-type (D), repo>dRas85DV12 alone (E), or repo>dPTENdsRNA alone (F). Compared to wild-type (D), repo>dEGFRwild-type (H) brains show reduced neurons (HRP, low intensity blue), which renders glia more densely packed and the entire brain smaller than normal. However, repo>dEGFRwild-type brains (H) do not show a substantial increase the number of glia compared to wild-type (D). Brains of repo>dEGFRElp 2nd instar larvae (I) show substantial neuron loss, brain malformation, and excess glia for that stage of development. Genotypes: (A) UAS-dEGFRλ UAS-dp110CAAX/+; repo-Gal4 UAS-CD8GFP/+ (B) UAS-CD8GFP/+; repo-Gal4/UAS-dRas85DV12 UAS-dPTENdsRNA (C) UAS-dp110CAAX; repo-Gal4 UAS-CD8GFP/UAS-dRafgof (D) repo-Gal4 UAS-CD8GFP/+ (E) repo-Gal4 UAS-CD8GFP/UAS-dRas85DV12 (F) UAS-CD8GFP/+; repo-Gal4/UAS-dPTENdsRNA (G) UAS-dEGFRλ/+; repo-Gal4 UAS-CD8GFP/+ (H) UAS-CD8GFP/+; repo-Gal4/UAS-dEGFRwild-type (I) UAS-CD8GFP/+; repo-Gal4/UAS-dEGFRElp. (10.31 MB TIF) [file pgen.1000374.s002.tif]

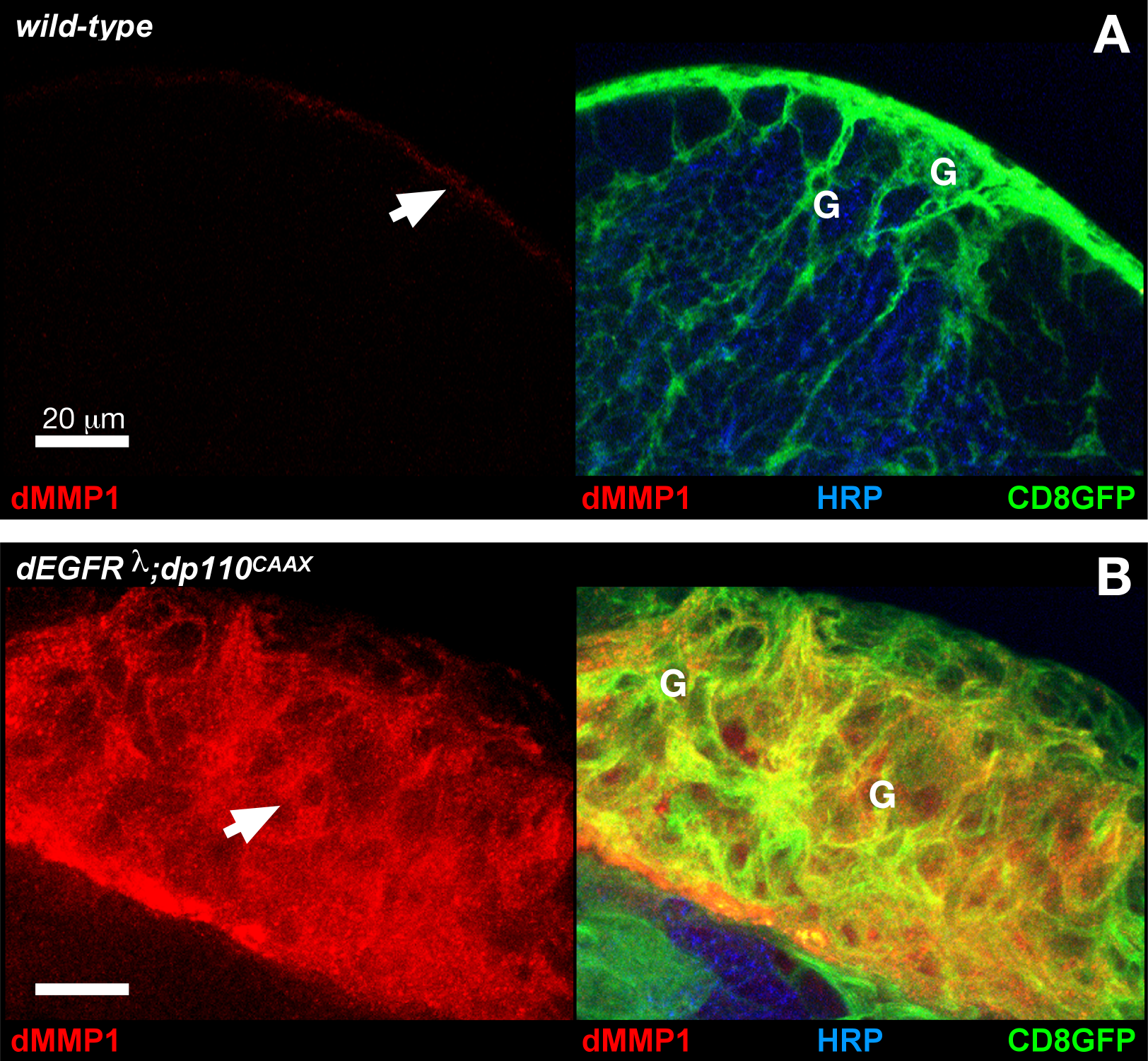

Supplement: Figure S3 — dMMP1 expression in wild-type and dEGFRλ;dp110CAAX glia. (A,B) 3rd instar larval brains. Frontal sections, showing medial regions enriched for proliferating glia. Anterior up; midline to left. 4.5 µm optical projections, matched in scale. 20 µm scale bars. Expression of the active form of dMMP1 (red) in wild-type (A) and repo>dEGFRλ;dp110CAAX brains (B), shown alone (left panels) and overlaid with an HRP (blue) neuronal label and a CD8GFP (green) glial label (right panels). In wild-type brains, glia (‘G’) rarely express active dMMP1, although some glia on the surface of the brain show low levels of dMMP1 staining (arrow). In repo>dEGFRλ;dp110CAAX brains (B), some neoplastic glia (‘G’) express active dMMP1 (red in right panel, yellow in overlay), which is largely membrane-localized in individual cells (arrow). Genotypes: (A) UAS-CD8GFP/+; repo-Gal4/+ (B) UAS-dEGFRλ UAS-dp110CAAX/+; UAS-CD8GFP/+; repo-Gal4/+. (3.93 MB TIF) [file pgen.1000374.s003.tif]

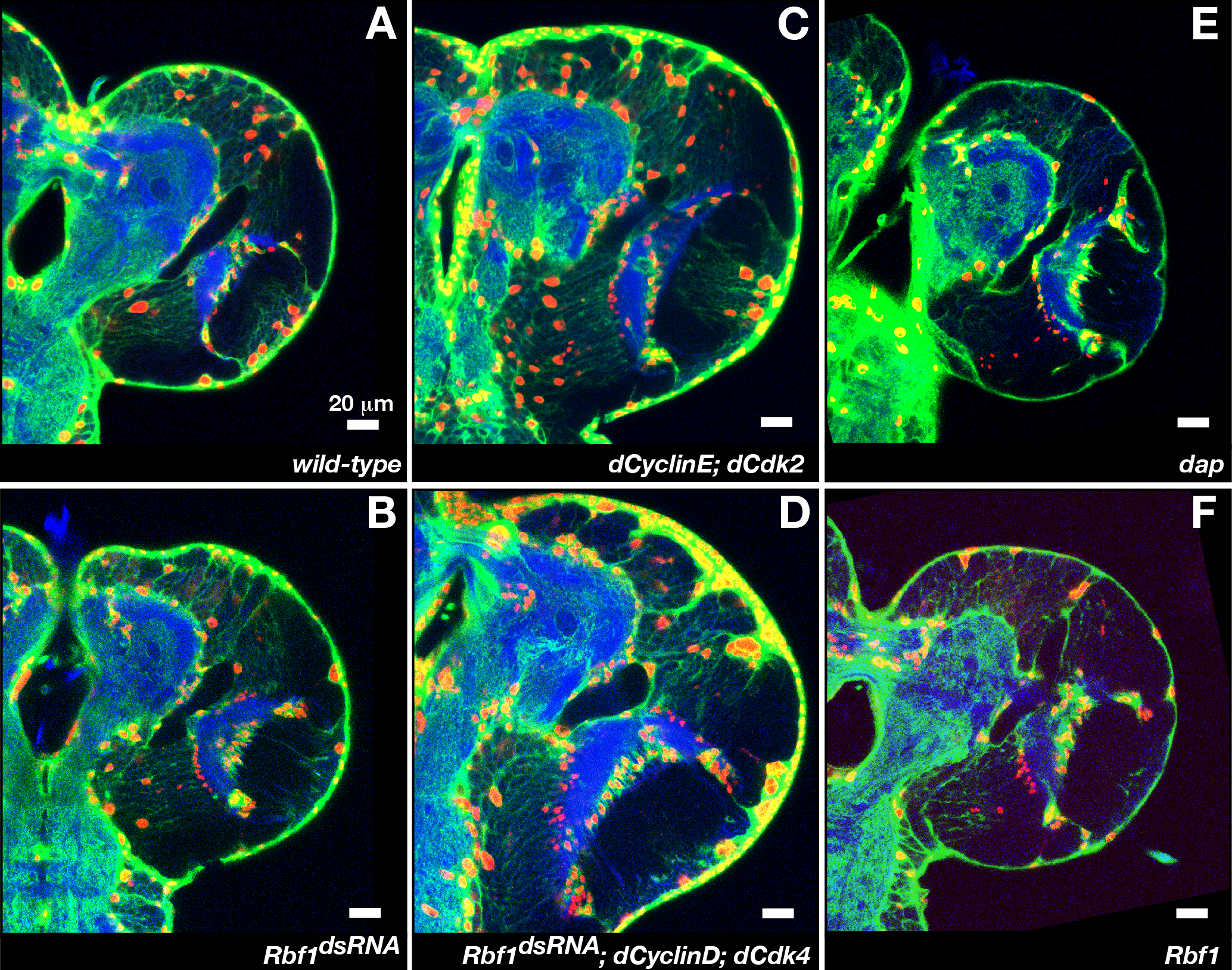

Supplement: Figure S4 — Glial-specific Rbf1 knock-down or overexpression of G1 Cyclin-Cdks, Dap, and Rbf1 affects glial proliferation. (A–F) 2 µm optical sections of larval brain hemispheres from wandering 3rd instar larvae displayed at the same scale. 20 µm scale bars. Frontal sections; midway through brains. Anterior up; midline to left. Glial cell nuclei are labeled with Repo (red). Glial cell bodies and membranes are labeled with CD8GFP (green) driven by repo-Gal4. HRP counter-staining (blue) reveals neuropil at high intensity and some cell bodies of neurons and neuronal precursors at low intensity. Rbf1 knock-down in repo>Rbf1dsRNA (B) does not significantly alter glial cell numbers (red nuclei) compared to wild-type (A). Ectopic expression of dCyclinE-dCdk2 (C) or dCyclinD-dCdk4 in repo-Gal4-glia promotes an approximate doubling of glial cells numbers (red nuclei), even when combined with Rbf1dsRNA (D). Continuous expression of Dap (E) or Rbf1 (F) in otherwise wild-type repo-Gal4-glia substantially reduces glial cell numbers, showing that repo-Gal4 glia normally undergo proliferation controlled by dCyclinE-dCdk2 and Rbf1-E2F1. Genotypes: (A) UAS-CD8GFP/+; repo-Gal4/+ (B) UAS-CD8GFP/+; repo-Gal4/UAS-Rbf1dsRNA (C) UAS-CD8GFP/+; repo-Gal4/UAS-dCyclinE UAS-dCdk2 (D) UAS-CD8GFP/UAS-dCyclinD UAS-dCdk4; repo-Gal4/UAS-Rbf1dsRNA (E) UAS-dap/+; UAS-CD8GFP/+; repo-Gal4/+ (F) UAS-CD8GFP/+; repo-Gal4/UAS-Rbf1. (8.27 MB TIF) [file pgen.1000374.s004.tif]

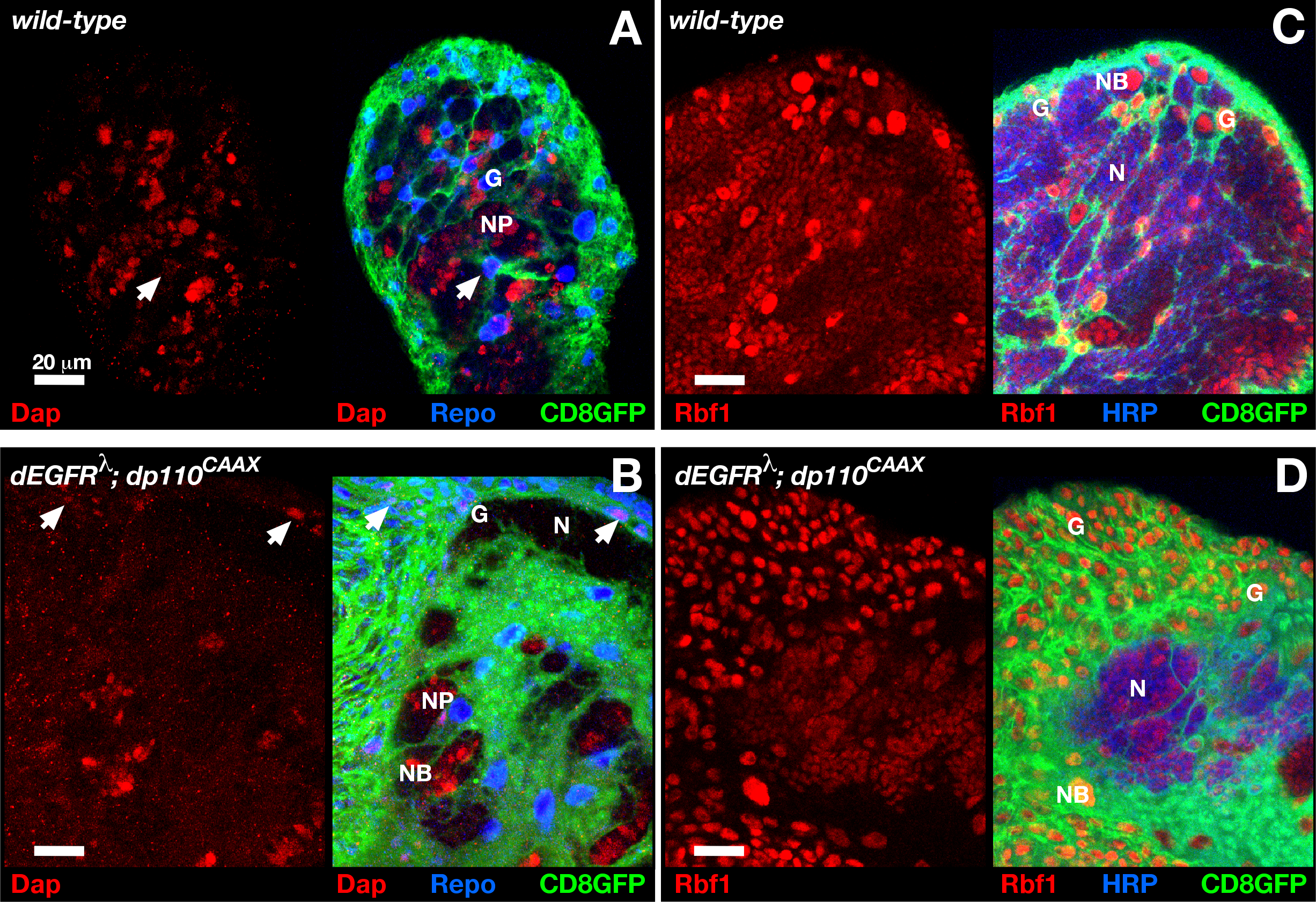

Supplement: Figure S5 — Dap and Rbf1 expression in wild-type and dEGFRλ;dp110CAAX glia. 3rd instar larval brains. Frontal sections, showing superficial dorsal regions enriched for Dap- and Rbf1-expressing cells. Anterior up; midline to left. 7 µm optical projections, all matched in scale. 20 µm scale bars. (A,B) Dap expression (red) in wild-type (A) and repo>dEGFRλ;dp110CAAX brains (B), shown alone (left panels) and overlaid with Repo (blue) and repo>CD8GFP (green) glial markers (right panels). In both genotypes, Dap is primarily expressed in neuroblasts (‘NB’) and ganglion mother cell neuronal precursors (‘NP’), rarely in glia (‘G’), and almost never in neurons (‘N’). In repo>dEGFRλ;dp110CAAX brains, Dap is rarely expressed in glia as seen by the lack of substantial overlap between Dap expression and glial markers (B, right panel). White arrows denote rare Dap-positive glia in both wild-type and repo>dEGFRλ;dp110CAAX brains; these glia show lower levels of Dap protein than neighboring neuroblasts and neuronal precursors. Dap-positive glia were counted in 3 repo>dEGFRλ;dp110CAAX brains and an average of 5% of all glia expressed Dap (87 glia expressed Dap out of 1738 total glia counted). (C–D) Rbf1 expression (red) in wild-type (E) and repo>dEGFRλ;dp110CAAX brains (F), shown alone (left) and overlaid (right) with the CD8GFP (green) glial marker and HRP (blue) neuronal marker. In both genotypes, Rbf1 is highly expressed in glia (‘G’) and neuroblasts (‘NB’), which were identified by their characteristic positions and large cell bodies. Neurons (‘N’) show lower expression. Genotypes: (A,C) UAS-CD8GFP/+; repo-Gal4/+ (B,D) UAS-dEGFRλ UAS-dp110CAAX/+; UAS-CD8GFP/+; repo-Gal4/+. (7.96 MB TIF) [file pgen.1000374.s005.tif]

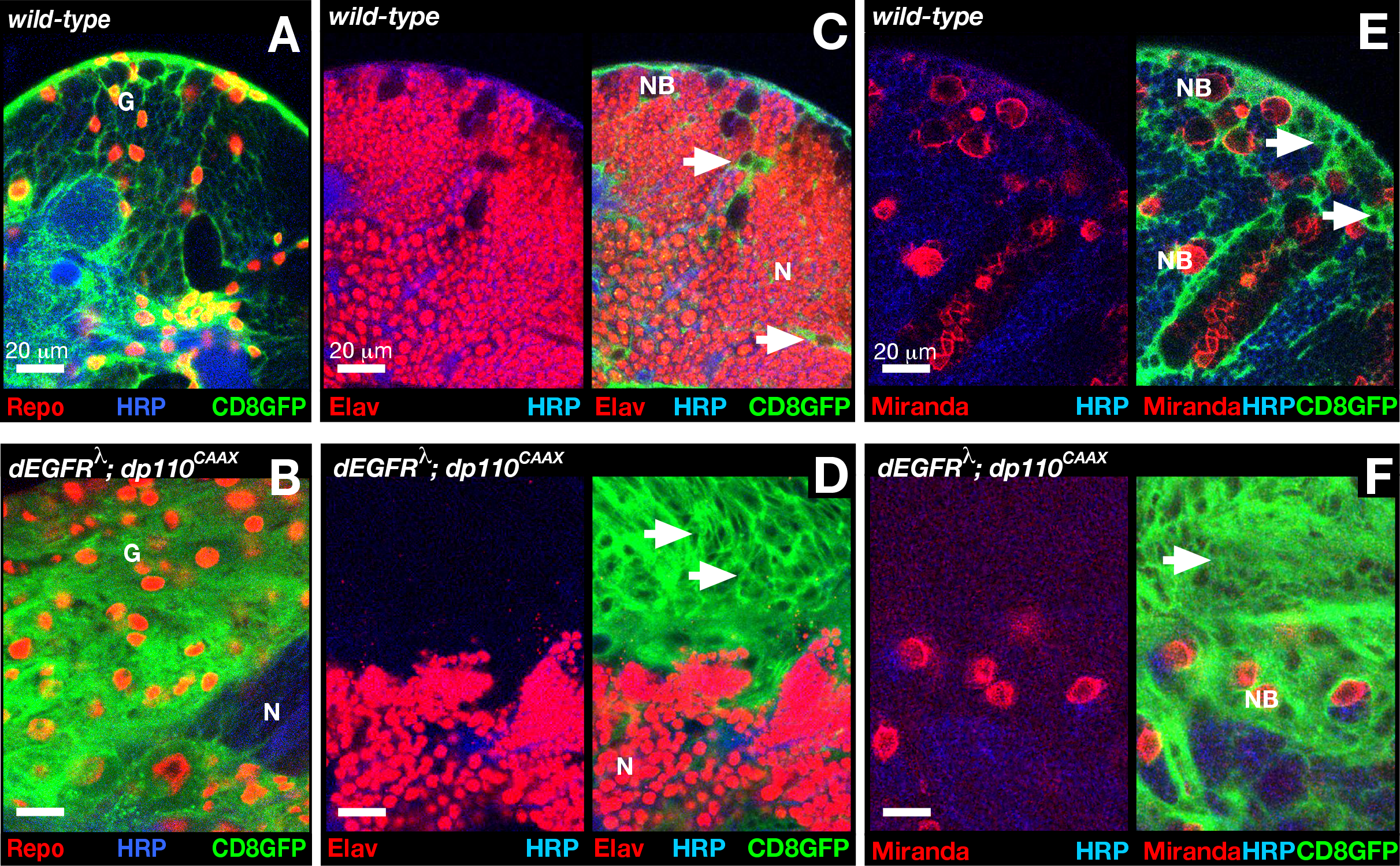

Supplement: Figure S6 — dEGFRλ; dp110CAAX glia do not express neuronal and neuroblast markers. Late 3rd instar larval brains. Medial anterior regions shown. Anterior up; midline to left. 2 µm optical sections. CD8GFP (green), driven by the repo-Gal4 driver, labels glial cell bodies and membranes. An HRP counter-stain (blue) reveals neuropil at high intensity and cell bodies of neurons and some neuronal precursors at low intensity. (A,B) Repo expression (red) in glial cell nuclei in wild-type (A) and repo>dEGFRλ;dp110CAAX brains (B). In wild-type brains, neuronal fibers and neurons (blue) are enveloped by glial processes (green) to give the brain a honeycombed appearance. In repo>dEGFRλ;dp110CAAX brains, neurons (‘N’) lack GFP or Repo expression, in contrast to glia (‘G’). (C–D) Elav expression (red) in wild-type (C) and repo>dEGFRλ;dp110CAAX brains (D), shown alone (left panels) and overlaid (right panels) with the CD8GFP (green) glial marker and HRP (blue) neuronal marker. In both genotypes, Elav is highly expressed in neurons (‘N’) but absent from glia (green cell bodies indicated by white arrows). (E–F) Miranda expression (red) in wild-type (E) and repo>dEGFRλ;dp110CAAX (F) brains, shown alone (left panels) and overlaid (right panels) with CD8GFP (green) glial marker and HRP (blue) neuronal marker. In both genotypes, Miranda is highly expressed in neuroblasts (‘NB’), which are identified by their characteristic positions and large cell bodies, but is absent from glia (green cell bodies, indicated by white arrows). Genotypes: (A,C,E) UAS-CD8GFP/+; repo-Gal4/+ (B,D,F) UAS-dEGFRλ UAS-dp110CAAX/+; UAS-CD8GFP/+; repo-Gal4/+. (9.24 MB TIF) [file pgen.1000374.s006.tif]

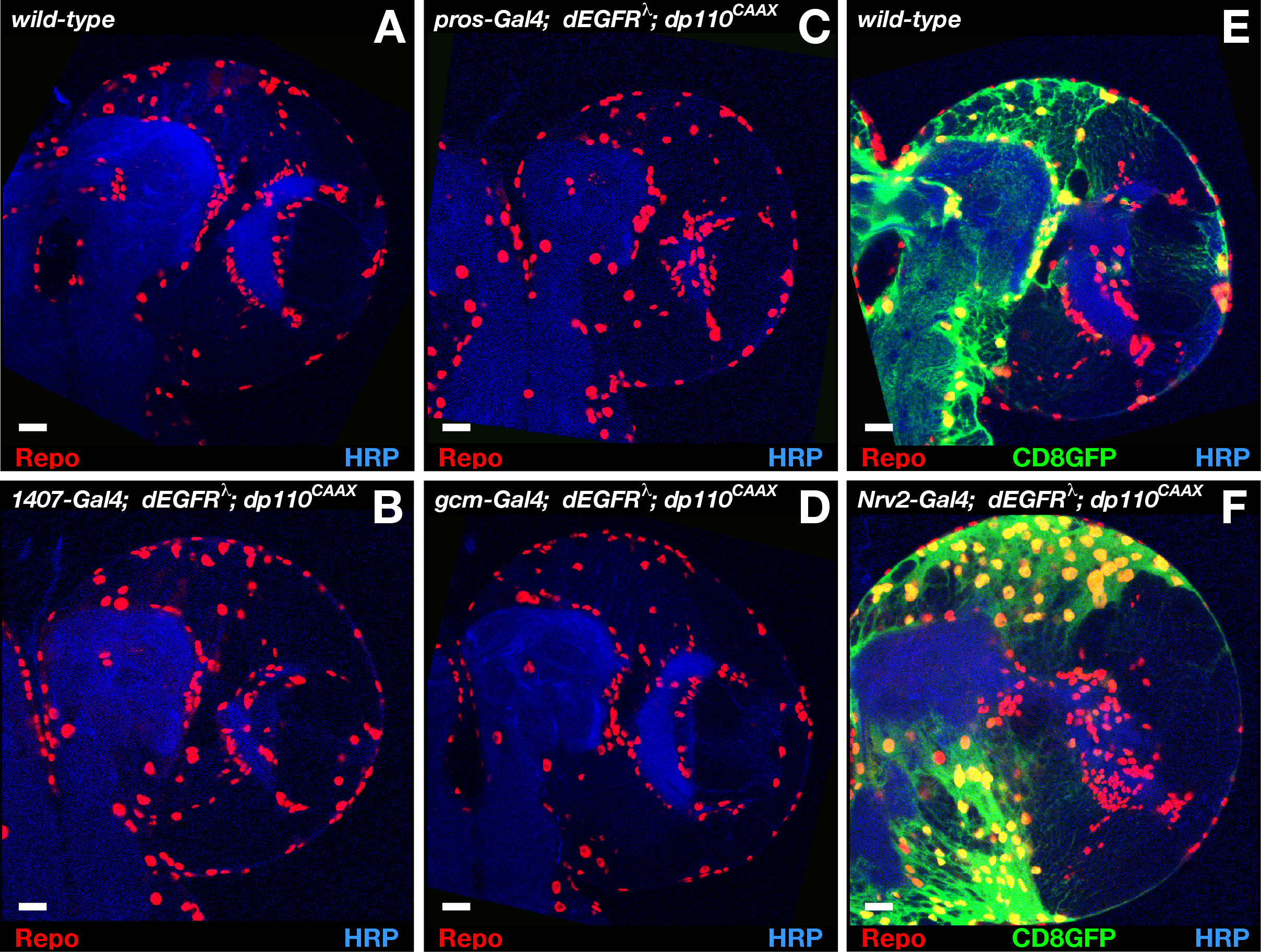

Supplement: Figure S7 — dEGFRλ and dp110CAAX do not induce neoplasia from neurons, neuroblasts, or certain glia. Late 3rd instar larval brain hemispheres. Medial anterior regions shown. Anterior up; midline to left. 3.5 µm optical projections. Repo expression (red) in glial cell nuclei in all genotypes. An HRP counter-stain (blue) reveals neuropil at high intensity and cell bodies of neurons and some neuronal precursors at low intensity, and this varies slightly according to variance in exact section plane. (A–D) Wild-type (A), dEGFRλ; dp110CAAX overexpressed using the 1407-Gal4 neuroblast driver (B), dEGFRλ; dp110CAAX overexpressed using the pros-Gal4 neural driver (C), and dEGFRλ;dp110CAAX overexpressed using the gcm-Gal4 embryonic glial driver (D). In all cases, dEGFRλ;dp110CAAX overexpression did not induce neoplastic overgrowth of glial or neuronal cell types, as determined by brain size and cell-type specific stains. (E,F) Cytoplasmic GFP (green), driven by the Nrv2-Gal4 driver, labels cell bodies and cytoplasmic processes, as seen in wild-type (E). dEGFRλ;dp110CAAX overexpressed using the Nrv2-Gal4 glial driver (B). Nrv2-Gal4 is expressed by post-mitotic cortex glia, which proliferate somewhat in response to dEGFRλ;dp110CAAX to cause slight brain enlargement, but do not become neoplastic like repo>dEGFRλ;dp110CAAX glia. Genotypes: (A) +/CyO (B) UAS-dEGFRλ UAS-dp110CAAX/+; 1407-Gal4/UAS-Gal4 (C) UAS-dEGFRλ UAS-dp110CAAX/+; UAS-Gal4/+; pros-Gal4/+ (D) UAS-dEGFRλ UAS-dp110CAAX/+; gcm-Gal4/+ (E) Nrv2-Gal4 UAS-GFP/+; Nrv2-Gal4 UAS-GFP/+ (F) UAS-dEGFRλ UAS-dp110CAAX/+; Nrv2-Gal4 UAS-GFP/UAS-Gal4; Nrv2-Gal4 UAS-GFP/+. (7.96 MB TIF) [file pgen.1000374.s007.tif]

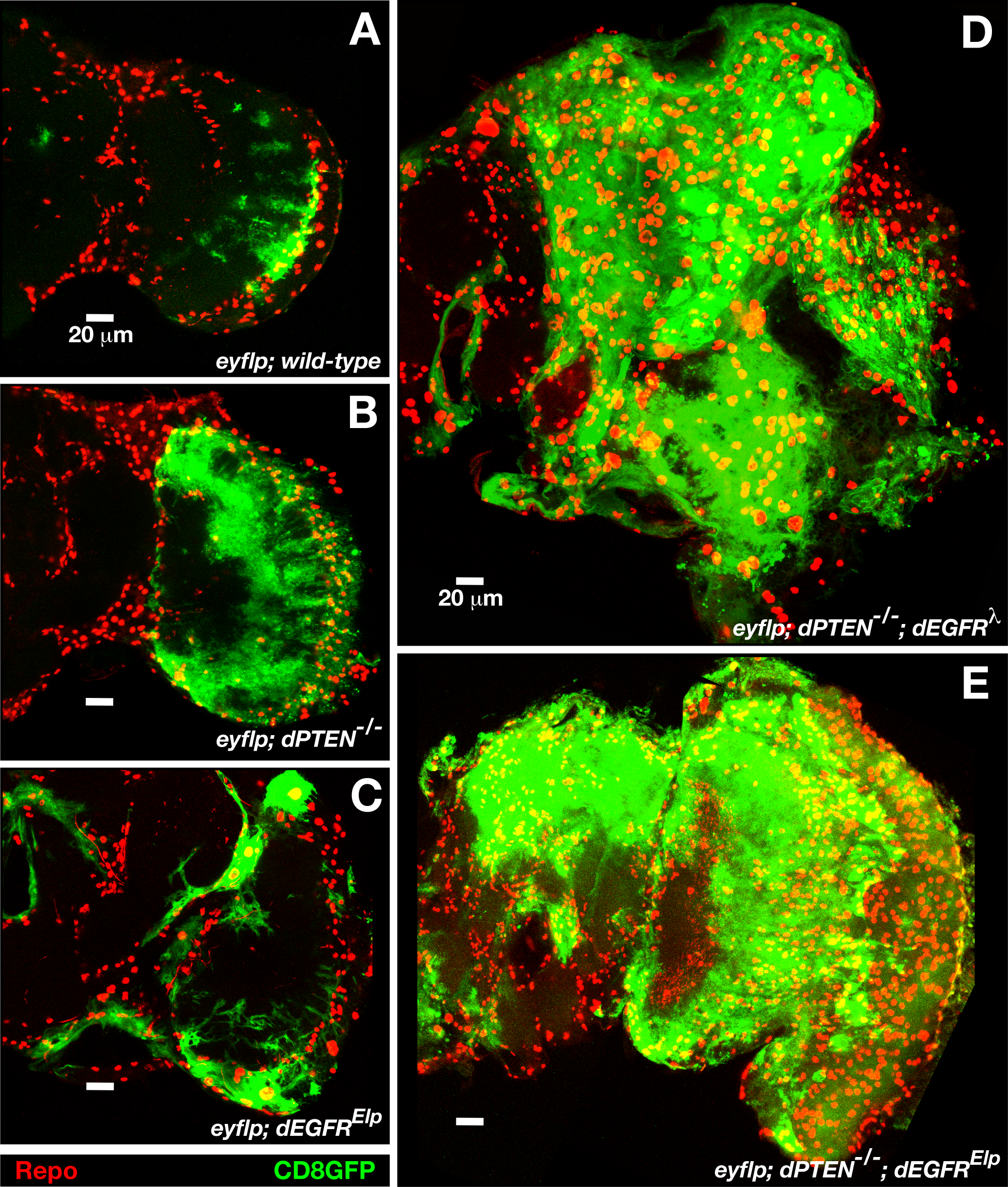

Supplement: Figure S8 — Coactivation of EGFR and PI3K in glial progenitors creates invasive neoplastic glia.(A–E) FLP/FRT clones in adult brains derived from a population of ey-FLP and repo-Gal4-expressing cells. CD8GFP (green) marks cell bodies and membranes of glial clones derived by FLP/FRT mitotic recombination (see text). Repo (red) marks all glial cell nuclei, in both clones and surrounding normal tissue. 8.5 µm confocal optical sections through brains of similarly aged adults, all matched to scale. 20 µm scale bars. Each panel shows half brains, including a whole optic lobe and adjacent central brain. dEGFRElp (C) and dPTEN−/− (J) clones are composed of 2–5-fold more cells than wild-type controls (H). In contrast, dEGFRλ;dPTEN−/− (D) and dEGFRElp;PTEN−/− double mutant clones form large tumors visible in adult brains. As with hs-FLP clones, dEGFRλ;dPTEN−/− and dEGFRElp;dPTEN−/− clones are less cellular and more invasive than dRas85DV12;PTEN−/− clones (see Figure 4). Genotypes: (A) ey-flp/+; FRT40A tubGal80/FRT,40A; repo-Gal4 UAS-CD8GFP/+ (B) ey-flp/+; FRT40A tubGal80/FRT40A PTEN2L117; repo-Gal4 UAS-CD8GFP/+ (C) ey-flp/+; FRTG13 tubGal80/FRTG13 UAS-CD8GFP; repo-Gal4/UAS-dEGFRElp (D) ey-flp/UAS- dEGFRλ; FRT40A tubGal80/FRT40A PTEN2L117; repo-Gal4 UAS-CD8GFP/UAS- dEGFRλ (E) ey-flp/+; FRT40A tubGal80/FRT40A PTEN2L117; repo-Gal4 UAS-CD8GFP/UAS-dEGFRElp. (7.84 MB TIF) [file pgen.1000374.s008.tif]

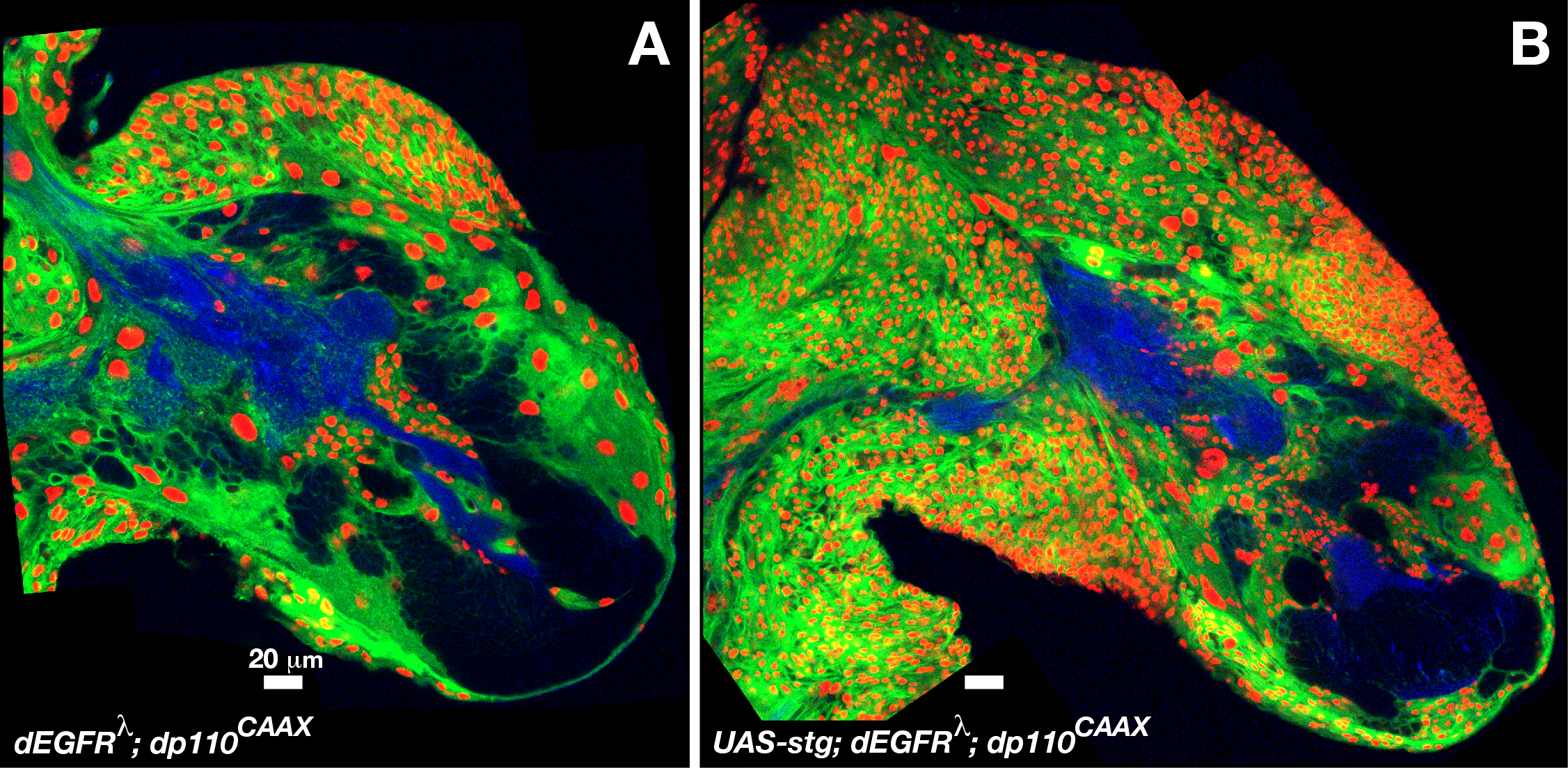

Supplement: Figure S9 — Stg overexpression exacerbates EGFR-PI3K glial neoplasia. 2 µm optical sections of larval brain hemispheres from late 3rd instar larvae, displayed at the same scale. 20 µm scale bars. Frontal sections, midway through brains. Anterior up; midline to left. Glial cell nuclei are labeled with Repo (red); glial cell bodies and membranes are labeled with CD8GFP (green) driven by repo-Gal4. An HRP counter-stain (blue) reveals neuropil at high intensity and neuronal cell bodies at low intensity. HRP stains varied between the two samples according to effects of mutant glia and slight variance in section plane. Stg co-overexpression with dEGFRλ;dp110CAAX (B) enhances neoplasia compared to dEGFRλ;dp110CAAX (A), leading to a dramatic increase in aberrant glia and yielding a phenotype similar to that of repo>Rbf1dsRNA;dEGFRλ;dp110CAAX (see Figure 8G). Genotypes: (A) UAS-dEGFRλ UAS-dp110CAAX/+; repo-Gal4 UAS-CD8GFP/+ (B) UAS-dEGFRλ UAS-dp110CAAX/+; UAS-stg/+; repo-Gal4 UAS-CD8GFP/+. (6.27 MB TIF) [file pgen.1000374.s009.tif]

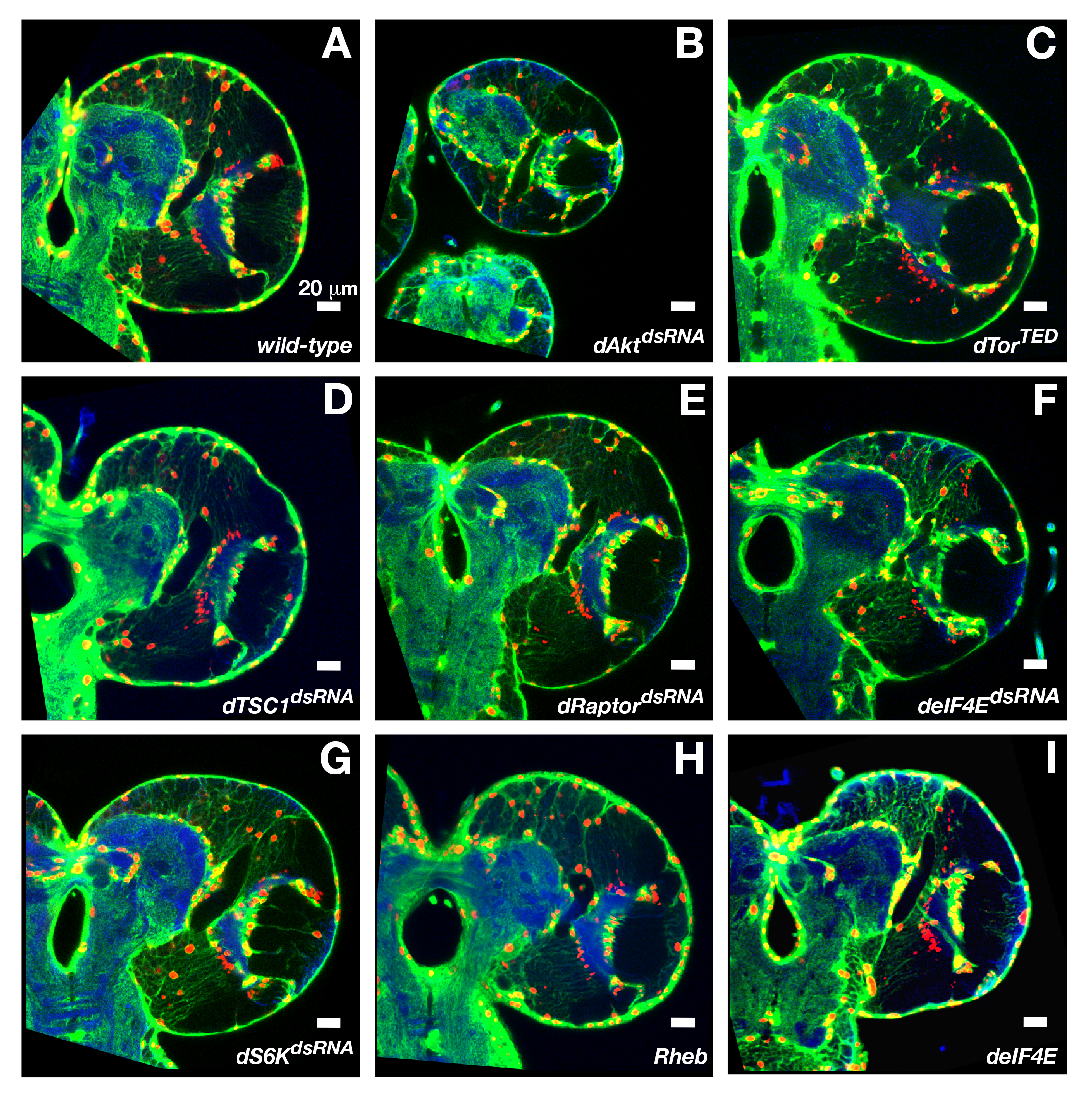

Supplement: Figure S10 — Knock-down and/or overexpression of dTor and dTor effectors. 2 µm optical sections of larval brain hemispheres from wandering 3rd instar larvae, approximately 130hr AED, all displayed at the same scale. 20 µm scale bars. Anterior up; midline to the left. Frontal sections; midway through brains. Repo (red) marks glial cell nuclei; CD8GFP (green), driven by repo-Gal4, labels glial cell bodies and membranes. An HRP-counter stain (blue) reveals neuropil (neuronal fiber tracts) at high intensity and some cell bodies of neurons and neuronal precursors at low intensity, and this varies slightly according to exact plane of section, brain orientation, and mutant phenotype. repo>dAktdsRNA brains (B) are smaller and have fewer glia than wild-type controls (A). All other genotypes have relatively normal sized brains. By gross examination, repo>TorTED (C), repo>dRaptordsRNA (E), repo>deIF4EdsRNA (F) and repo>dS6KdsRNA (G) display an estimated 10–20% reduction in glial cell numbers. repo>dTSC1dsRNA (D), repo>Rheb (H) and repo>deIF4E (I) brains contain normal numbers of glia. Genotypes: (A) repo-Gal4 UAS-CD8GFP/+ (B) UAS-CD8GFP/+; repo-Gal4/UAS-dAktdsRNA (C) UAS-TorTED/UAS-CD8GFP; repo-Gal4/+ (D) UAS-CD8GFP/+; repo-Gal4/UAS-dTSC1dsRNA (E) UAS-dRaptordsRNA/UAS-CD8GFP; repo-Gal4/+ (F) UAS-CD8GFP/+; repo-Gal4/UAS-deIF4EdsRNA (G) UAS-dS6KdsRNA; UAS-CD8GFP/+; repo-Gal4/+ (H) UAS-CD8GFP/+; repo-Gal4/UAS-Rheb (I) UAS-CD8GFP//UAS-deIF4E; repo-Gal4/+. (8.83 MB TIF) [file pgen.1000374.s010.tif]

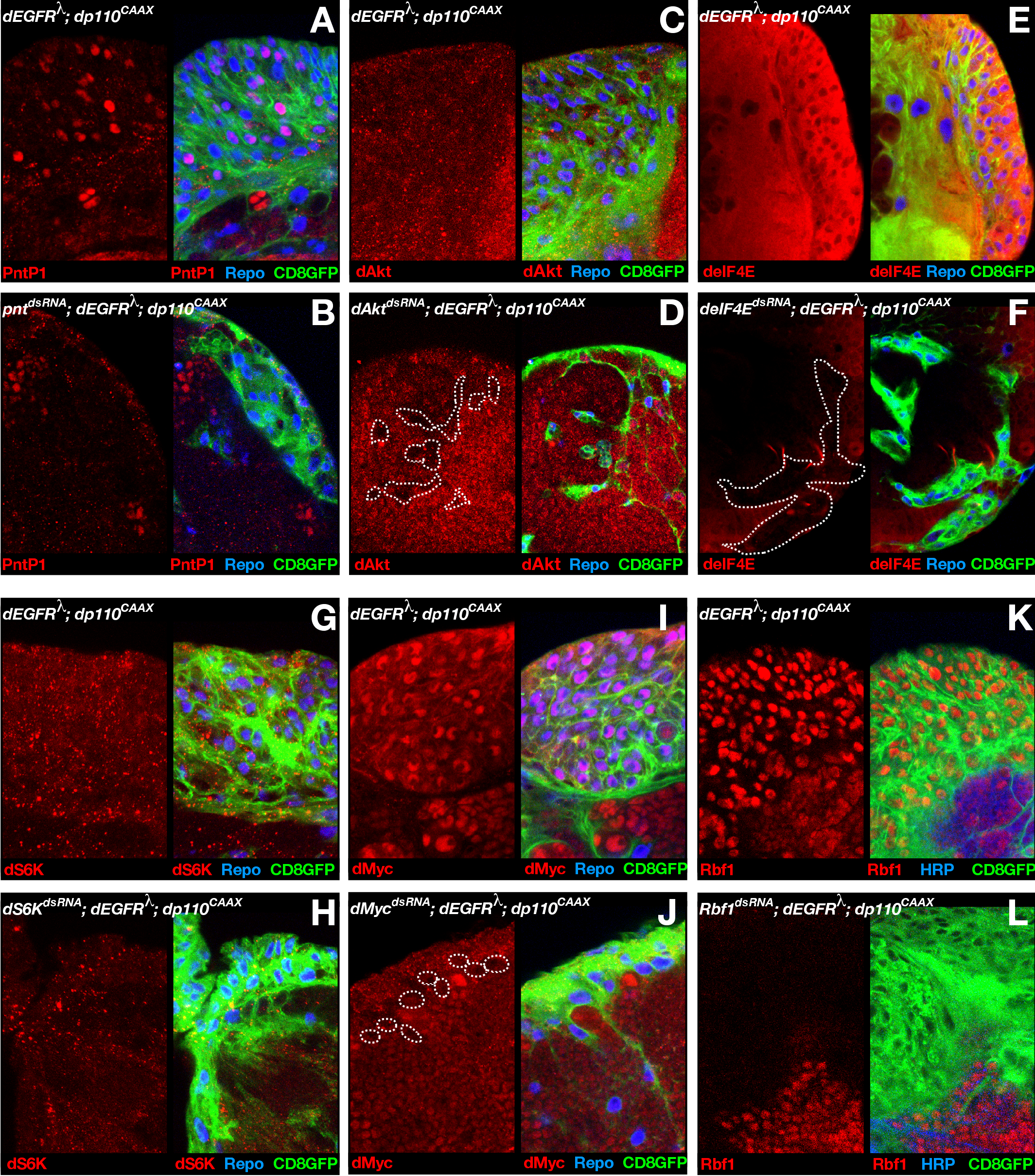

Supplement: Figure S11 — Validation of dsRNA constructs. 2 µm optical sections of larval brain hemispheres from wandering 3rd instar larvae. Frontal sections. Anterior up, midline to the left. Each individual staining pattern is shown alone (left panels) and with overlaid glial or neuronal markers (right panels). Repo (blue) in (A–J) marks glial cell nuclei. Glial cell bodies and membranes are labeled with CD8GFP (green) driven by repo-Gal4. In (K,L) an HRP counter-stain (blue) reveals neurons. Red marks histochemical stains for each indicated protein in repo>dEGFRλ;dp110CAAX (A,C,E,G,I,K) and repo>dEGFRλ;dp110CAAX with each indicated dsRNA construct (B,D,F,H,J,L). Each dsRNA construct was expressed with repo-Gal4 to yield glial-specific knock-down, which left protein expression in surrounding neuronal tissue intact. For the nuclear protein PntP1, knock-down was verified by the lack of glial-specific staining in the presence of the pntdsRNA (B), although PntP1 is present within neighboring neurons (‘N’). For dAkt, deIF4E, and dS6K, gene knock-down was confirmed using antibodies for total protein. Glial-specific reduction in gene expression by dAktdsRNA and deIF4EdsRNA is highlighted by white outlines in (D) and (F). dAkt protein is higher in neuronal tissue (‘N’) in both repo>dEGFRλ;dp110CAAX (C) and repo>dAktdsRNA;dEGFRλ;dp110CAAX (D). deIF4E is high in dEGFRλ;dp110CAAX glia (E) and low in neurons (E,F). repo>deIF4EdsRNA;dEGFRλ;dp110CAAX brains (F) have reduced glial eIF4E. Reduced glial dS6K protein, which is diffusely cytoplasmic, is observed in repo>dS6KdsRNA;dEGFRλ;dp110CAAX brains (H) compared to repo>dEGFRλ;dp110CAAX (G). Reduced glial dMyc expression in repo>dMycdsRNA;dEGFRλ;dp110CAAX brains (J) is noted by white outlines, and the absence of purple nuclei in overlay (J, right panel), relative to repo>dEGFRλ;dp110CAAX (I, right panel). dMyc is highly expressed in neuroblasts (‘NB’) in both samples (I, J), which do not express the dMycdsRNA. Glial Rbf1 protein is absent in repo>dRbf1d [file pgen.1000374.s011.tif]

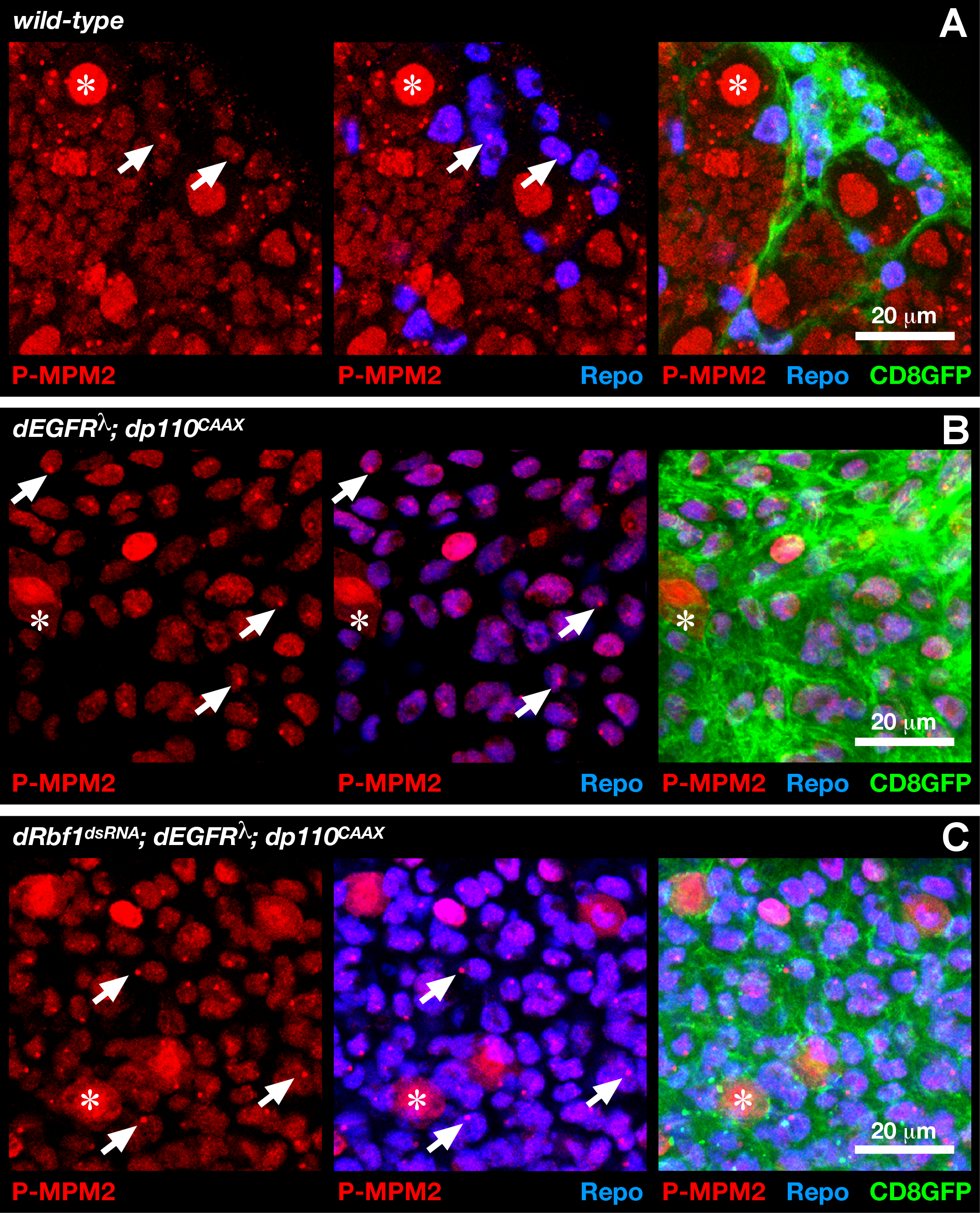

Supplement: Figure S12 — Phospho-MPM2 reveals increased S-phase and M-phase glia in repo>dRbf1dsRNA;dEGFRλ;dp110CAAX brains. (A–C) phospho-MPM2 expression (red) in wild-type brains (A), repo>dEGFRλ;dp110CAAX brains (B), and repo>dRbf1dsRNA;dEGFRλ;dp110CAAX brains (C). 20 µm scale bars. Anterior up, midline to the left. 6 µm optical projections showing representative superficial dorsal regions enriched for mitotic glia. Phospho-MPM2 shown alone (left panels), overlaid with the Repo (blue, middle panels), and CD8GFP (green, right panels) glial markers. Phospho-MPM2 nuclear foci are present in S-phase cells (arrows note examples). S-phase glia are clearly visible in the middle panel as purple cells with MPM2 foci, which appear enriched in repo>dRbf1dsRNA;dEGFRλ;dp110CAAX brains (C) relative to repo>dEGFRλ;dp110CAAX brains (B). In all genotypes, high levels of phospho-MPM2 is also expressed in mitotic cells (asterisks note examples). Mitotic glia showed low levels of Repo (middle panels), but are clearly GFP-positive (right panels), suggesting that Repo protein expression is reduced upon mitosis in glia. repo>dRbf1dsRNA;dEGFRλ;dp110CAAX brains (C) also show increased density of phospho-MPM2-positive mitotic glia compared to repo>dEGFRλ;dp110CAAX brains (B). In wild-type (A), the majority of phospho-MPM2-postive cells are not glial and are neuroblasts or neuronal precursors, as revealed by the lack of overlap between phospho-MPM2 staining (middle panel) and the Repo and CD8GFP markers. Genotypes: (A) repo-Gal4 UAS-CD8GFP/+ (B) UAS-dEGFRλ UAS-dp110CAAX/+; UAS-CD8GFP/+; repo-Gal4/+ (C) UAS-dEGFRλ UAS-dp110CAAX/+; repo-Gal4 UAS-CD8GFP/UAS-Rbf1dsRNA. (8.99 MB TIF) [file pgen.1000374.s012.tif]
