# Supplementary material for: A Drosophila Model for EGFR-Ras and PI3K-Dependent Human Glioma
Source: PLoS Genet. 2009 Feb 13;5(2):e1000374. doi: 10.1371/journal.pgen.1000374 (PMC2636203; doi:10.1371/journal.pgen.1000374)
Supplement: Table S1 — Overexpression of EGFR-Ras and PI3K-Akt pathways in Drosophila glia. (0.08 MB PDF) [file pgen.1000374.s013.pdf]

Table S1: Overexpression of EGFR-Ras and PI3K-Akt pathways in *Drosophila* glia

**Gene (UAS construct)**	***repo-Gal4* glial overexpression phenotypes**
$dEGFR^{\lambda}$<br>$dEGFR^{Elp}$<br>$dEGFR^{wild\text{-}type}$<br>$dRas85D^{V12}$<br>$dRaf^{gof}$	glial hyperplasia, CNS morphogenesis defects, lethal
$dp110^{CAAX}$<br>$dp110^{wild\text{-}type}$<br>$dPTEN^{dsRNA}$<br>*dAkt*	normal glial cell numbers-slight glial hyperplasia, viable
$dEGFR^{\lambda}$; $dp110^{CAAX}$<br>$dEGFR^{\lambda}$; *dAkt*<br>$dRas85D^{V12}$; $dp110^{CAAX}$<br>$dRas85D^{V12}$; $dPTEN^{dsRNA}$<br>$dRas85D^{V12}$; $dPTEN^{+/-}$<br>$dRas85D^{V12}$; *dAkt*<br>$dRaf^{gof}$; $dp110^{CAAX}$	glial neoplasia, neuronal loss, behavior defects, CNS morphogenesis defects including enlarged brain lobes and elongated ventral nerve cord, early lethality or prolonged larval phase before pupal lethality
$dEGFR^{\lambda}$; $dp110^{CAAX}$; $dRas85D^{N17}$	Suppression of neoplasia and other phenotypes, sometimes viable
$dEGFR^{\lambda}$; $dp110^{CAAX}$; $dPTEN^{wild\text{-}type}$	Suppression of neoplasia and other phenotypes, lethal

3rd instar larval brains for each genotype were scored for glial cell numbers, morphology, and position, when possible. 1st and 2nd instar brains were scored for genotypes that showed earlier lethality, including $dEGFR^{Elp}$ and $dRas85D^{V12}$; *dAkt*.
