# Supplementary material for: A Drosophila Model for EGFR-Ras and PI3K-Dependent Human Glioma
Source: PLoS Genet. 2009 Feb 13;5(2):e1000374. doi: 10.1371/journal.pgen.1000374 (PMC2636203; doi:10.1371/journal.pgen.1000374)
Supplement: Table S2 — Coactivation of EGFR and PI3K does not elicit neoplasia in all neural cell types. (0.08 MB PDF) [file pgen.1000374.s014.pdf]

Table S2: Co-activation of EGFR and PI3K does not elicit neoplasia in all neural cell types

**Neural cell type**	**Gal4 lines tested with *dEGFR*$^{\lambda}$*; dp110*^{CAAX}**
neuronal precursors, neurons	*elav-Gal4* [b], *scratch-Gal4* [a], *OK107-Gal4, Appl-Gal4* [a]
neuroblasts, neural precursors	*worniu-Gal4* [a], *1407-Gal4* [a, b]
mixed lineage neurons, neuroblasts, some glia	*pros-Gal4* [a, b]
differentiated glia	*Eaat-Gal4* [a], *Nrv2-Gal4* [a, c], *Mz97-Gal4* [a, b]
embryonic glia	*gcm-Gal4* (transient in glia)

For all *Gal4-UAS-dEGFR*$^{\lambda}$ *UAS-dp110*^{CAAX} combinations listed, 3rd instar larval brains were examined for expression of Repo protein (to count glial cell numbers) and Elav protein (to count neuronal cell numbers). Control crosses of each *Gal4* to *UAS-CD8GFP* were also performed to verify expression patterns.

[a] *UAS-dEGFR*$^{\lambda}$*; UAS-dp110*^{CAAX} combined with *UAS-Gal4* amplification
[b] lethality in larval or early pupal stages both with and without *UAS-Gal4* amplification
[c] lethality only with *UAS-Gal4* amplification
