# Supplementary material for: A Drosophila Model for EGFR-Ras and PI3K-Dependent Human Glioma
Source: PLoS Genet. 2009 Feb 13;5(2):e1000374. doi: 10.1371/journal.pgen.1000374 (PMC2636203; doi:10.1371/journal.pgen.1000374)
Supplement: Table S3 — Genetic analysis of Akt, Tor, Myc, and CyclinD-Cdk4 pathways in neoplastic and normal glia. (0.10 MB PDF) [file pgen.1000374.s015.pdf]

Table S3: Genetic analysis of Akt, Tor, Myc, and CyclinD-Cdk4 pathways in neoplastic and normal glia

**Functional genotype**	**Glial neoplasia/phenotypes relative to *repo>dEGFR*$^{\lambda}$*;dp110*^{CAAX}**
repo>dAkt^{dsRNA}*; dEGFR*$^{\lambda}$*; dp110*^{CAAX}	full suppression [a]
dAkt$^{04226/+}$*; repo>dEGFR*$^{\lambda}$*; dp110*^{CAAX}	partial suppression
repo>dFoxO$^{wild\text{-}type}$*; dEGFR*$^{\lambda}$*; dp110*^{CAAX}	partial suppression
repo>dFoxO^{SA}*; dEGFR*$^{\lambda}$ *;dp110*^{CAAX}	partial suppression [a]
repo>dTor^{TED}*; dEGFR*$^{\lambda}$*; dp110*^{CAAX}	full suppression [a]
dTor$^{2L7/l(2)k17004}$*; repo>dEGFR*$^{\lambda}$*; dp110*^{CAAX}	partial suppression
dSin1$^{e03756/l(2)Bsc11}$*; repo>dEGFR*$^{\lambda}$*; dp110*^{CAAX}	strong suppression [a]
dRictor$^{\Delta 2}$*; repo>dEGFR*$^{\lambda}$*; dp110*^{CAAX}	strong suppression
repo>dRaptor^{dsRNA}*; dEGFR*$^{\lambda}$*; dp110*^{CAAX}	strong suppression [a]
repo>dS6K^{dsRNA}*; dEGFR*$^{\lambda}$*; dp110*^{CAAX}	strong suppression [a]
repo>deIF4E^{dsRNA}*; dEGFR*$^{\lambda}$*; dp110*^{CAAX}	strong suppression [a]
repo>d4EBP; dEGFR$^{\lambda}$*; dp110*^{CAAX}	strong suppression [a]
repo>dTSC1^{dsRNA}*; dEGFR*$^{\lambda}$*; dp110*^{CAAX}	enhancement [a]
repo>dMyc^{dsRNA}*; dEGFR*$^{\lambda}$*; dp110*^{CAAX}	strong suppression [b]
dMyc$^{P0/+}$*; repo>dEGFR*$^{\lambda}$*; dp110*^{CAAX}	moderate suppression, sometimes viable [b]
dMyc$^{4/+}$*; repo>dEGFR*$^{\lambda}$*; dp110*^{CAAX}	moderate suppression
repo>dMax^{dsRNA}*; dEGFR*$^{\lambda}$*; dp110*^{CAAX}	strong suppression [b]
dCdk4$^{3/k06503}$*; repo>dEGFR*$^{\lambda}$*; dp110*^{CAAX}	strong suppression [b]
repo>dCdk4^{dsRNA}*; dEGFR*$^{\lambda}$*; dp110*^{CAAX}	strong suppression
repo>dCyclinD^{dsRNA}*; dEGFR*$^{\lambda}$*; dp110*^{CAAX}	partial suppression
repo>Rbf1^{dsRNA}*; dEGFR*$^{\lambda}$*; dp110*^{CAAX}	synergistic enhancement [c]
repo>dEGFR$^{\lambda}$*; deIF4E*	similar to dEGFR$^{\lambda}$ alone
repo>dEGFR$^{\lambda}$*; dS6K*^{act}	similar to dEGFR$^{\lambda}$ alone
repo>dEGFR$^{\lambda}$*; dMyc*	neoplastic glia [c]
repo>dEGFR$^{\lambda}$*; dCyclinD; dCdk4*	weak glial neoplasia [c]
repo>dEGFR$^{\lambda}$*;Rbf1*^{dsRNA}	glial overproliferation [c]
repo>dAkt^{dsRNA}	reduced glia, small brain, pupal lethal [d]
repo>dFoxo$^{wild\text{-}type}$	early lethal
repo>dTor^{TED}	reduced glia, small brain, lethal [d]
dSin1$^{e03756/l(2)Bsc11}$	grossly normal glia, viable [a]
repo>dRaptor^{dsRNA}	slight glial hypoplasia [d]
repo>dS6K^{dsRNA}	slight glial hypoplasia [d]
repo>deIF4E^{dsRNA}	slight glial hypoplasia, pupal lethal [d]
repo>dTSC1^{dsRNA}	not neoplastic [d]
repo>Rheb	viable
repo>deIF4E	viable

repo>dS6K^{act}	not neoplastic
repo>dMyc^{dsRNA}	reduced glia, small brain, larval lethal
dCdk4$^{3/k06503}$	grossly normal glia, viable [b]
repo>dMyc	glial polyploidy, some excess glia, not neoplastic, viable
repo> dCyclinD; dCdk4	slight increase in glia, not neoplastic [e]
repo> Rbf1^{dsRNA}	slight increase in glia, not neoplastic [e]

3rd instar larval brains were examined for all genotypes, except *repo>dFoxO* since overexpression of either dFoxO^{SA} or dFoxO$^{wild\text{-}type}$ caused early lethality.

[a] representative brain hemisphere shown in Figure 6, see legend for full genotype
[b] representative brain hemisphere shown in Figure 7, see legend for full genotype
[c] representative brain hemisphere shown in Figure 8, see legend for full genotype
[d] representative brain hemisphere shown in Figure S10, see legend for full genotype
[e] representative brain hemisphere shown in Figure S4, see legend for full genotype
